# Primary prevention of acute cardiovascular events by influenza vaccination: an observational study

**DOI:** 10.1093/eurheartj/ehac737

**Published:** 2022-12-20

**Authors:** Jennifer A Davidson, Amitava Banerjee, Ian Douglas, Clémence Leyrat, Richard Pebody, Helen I McDonald, Emily Herrett, Harriet Forbes, Liam Smeeth, Charlotte Warren-Gash

**Affiliations:** Department of Non-Communicable Disease Epidemiology, London School of Hygiene and Tropical Medicine, Keppel Street, London WC1E 7HT, UK; Institute of Health Informatics, University College London, 222 Euston Road, London NW1 2DA, UK; Department of Non-Communicable Disease Epidemiology, London School of Hygiene and Tropical Medicine, Keppel Street, London WC1E 7HT, UK; Department of Non-Communicable Disease Epidemiology, London School of Hygiene and Tropical Medicine, Keppel Street, London WC1E 7HT, UK; Department of Medical Statistics, London School of Hygiene and Tropical Medicine, Keppel Street, London WC1E 7HT, UK; Institute of Epidemiology and Health Care, University College London, 1-19 Torrington Place, London NW1 2DA, UK; National Institute for Health Research Health Protection Research Unit in Immunisation, London School of Hygiene and Tropical Medicine in partnership with the UK Health Security Agency, Keppel Street, London WC1E 7HT, UK; Department of Infectious Disease Epidemiology, London School of Hygiene and Tropical Medicine, Keppel Street, London WC1E 7HT, UK; Department of Non-Communicable Disease Epidemiology, London School of Hygiene and Tropical Medicine, Keppel Street, London WC1E 7HT, UK; Population Health Sciences, Bristol Medical School, University of Bristol, Queens Road, Bristol BS8 1QU, UK; Department of Non-Communicable Disease Epidemiology, London School of Hygiene and Tropical Medicine, Keppel Street, London WC1E 7HT, UK; Department of Non-Communicable Disease Epidemiology, London School of Hygiene and Tropical Medicine, Keppel Street, London WC1E 7HT, UK

**Keywords:** Influenza vaccine, Cardiovascular complications, hypertension, QRISK

## Abstract

**Aims:**

Previous studies show a reduced incidence of first myocardial infarction and stroke 1–3 months after influenza vaccination, but it is unclear how underlying cardiovascular risk impacts the association.

**Methods and results:**

The study used linked Clinical Practice Research Datalink, Hospital Episode Statistics Admitted Patient Care and Office for National Statistics mortality data from England between 1 September 2008 and 31 August 2019. From the data, individuals aged 40–84 years with a first acute cardiovascular event and influenza vaccination occurring within 12 months of each September were selected. Using a self-controlled case series analysis, season-adjusted cardiovascular risk stratified incidence ratios (IRs) for cardiovascular events after vaccination compared with baseline time before and >120 days after vaccination were generated. 193 900 individuals with a first acute cardiovascular event and influenza vaccine were included. 105 539 had hypertension and 172 050 had a QRISK2 score ≥10%. In main analysis, acute cardiovascular event risk was reduced in the 15–28 days after vaccination [IR 0.72 (95% CI 0.70–0.74)] and, while the effect size tapered, remained reduced to 91–120 days after vaccination [0.83 (0.81–0.88)]. Reduced cardiovascular events were seen after vaccination among individuals of all age groups and with raised and low cardiovascular risk.

**Conclusions:**

Influenza vaccine may offer cardiovascular benefit among individuals at varying cardiovascular risk. Further studies are needed to characterize the populations who could derive the most cardiovascular benefits from vaccination.

## Structured Graphical Abstract

**Structured Graphical Abstract:**
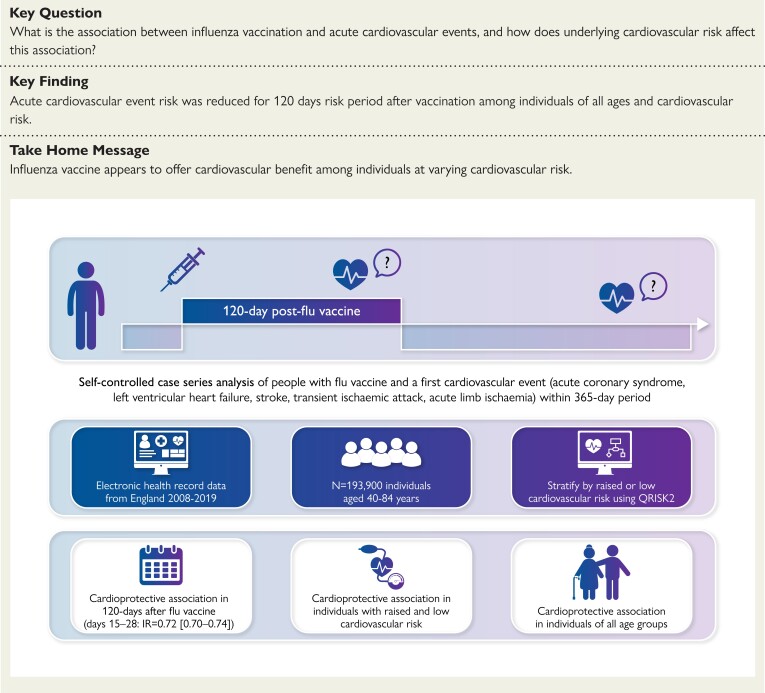
Graphical summary of the main findings of the self-controlled case series analysis conducted using electronic health records from the Clinical Practice Research Datalink and the Hospital Episode Statistics investigating the association between influenza vaccination and reduced incidence of first acute cardiovascular events.

**See the editorial comment for this article ‘The ideal vaccine to prevent cardiovascular disease’, by O. Fröbert et al.,https://doi.org/10.1093/eurheartj/ehac826.**

## Introduction

Annual trends in acute cardiovascular events, such as myocardial infarction (MI) and cardiovascular mortality, mirror influenza seasonality. A population-level association between influenza circulation and cardiovascular events exists after controlling for incidence trends, seasonality, and environmental factors.^1,2^ Previous studies show MI and stroke incidences are up to six times higher in early time periods after clinically diagnosed influenza-like illness or laboratory-confirmed influenza virus infection.^3,4^ Several underlying mechanisms may explain influenza-triggered cardiovascular events: influenza may directly affect the vascular cells or induce haemodynamic, inflammatory, and pro-coagulant processes.^5^

Influenza-related complications and mortality are common among older individuals and those with underlying health conditions, such as established cardiovascular disease (CVD).^6^ Before the COVID-19 pandemic in England, like many high-income countries, long-standing policy recommended influenza vaccination for everyone aged ≥65 years and adults aged <65 years with an underlying health condition (‘clinical risk group’).^7,8^ Since the winter of 2020–21, vaccine policy recommendations in England have been extended to include all adults aged ≥50 years. Widespread uptake of influenza vaccine aims to protect individuals at risk of severe illness and reduce health service pressure during winter months by lessening influenza morbidity. Reducing winter health service pressure has been critically important during the COVID-19 pandemic, which has caused substantial health system burden as well as significant morbidity and mortality. In England, influenza vaccine uptake in individuals aged ≥65 years is routinely high at nearly 75%^9^ but low in adults aged <65 years in a clinical risk group,^9,10^ and was also only 35% among the newly recommended group of people aged 50–64 years and not in a clinical risk group in 2020–21.^9^ The vaccine uptake seen in England is far higher than that in many other European countries.^8^

Influenza as a trigger of cardiovascular complications provides a potential target for CVD prevention by vaccination. Three meta-analyses of secondary prevention randomized controlled trials (RCTs) among people with chronic heart disease found a significant reduction in cardiovascular mortality (55%)^11^ and cardiovascular complications (34–36%)^12,13^ after influenza vaccination. Recent RCTs continue to evaluate the cardiovascular benefits of vaccination among individuals with CVD.^14–18^ Trial results suggest the cardiovascular protection provided by influenza vaccine is comparable with other secondary CVD prevention strategies.^19^ However, there are no RCTs which have examined use of influenza vaccine for primary CVD prevention. Results from observational studies are mixed but suggest a reduction in the relative incidence of first MI and stroke 1–3 months after vaccination.^20–23^ Therefore, influenza vaccine may also have a role in primary CVD prevention.^24^ People with raised cardiovascular risk, e.g. due to hypertension, but without established CVD, are not specifically recommended to receive the influenza vaccine in England. Recent analyses found an increased incidence of cardiovascular complications after acute respiratory infections, including pneumonia, influenza, and COVID-19, among adults with raised cardiovascular risk.^25,26^

Our current study aimed to investigate the association between influenza vaccination and acute cardiovascular events, considering individual cardiovascular risk, using the self-controlled case series (SCCS) method^27^ with linked electronic health records from England.

## Methods

### Data sources

We used linked English anonymized data from the Clinical Practice Research Datalink (CPRD) Aurum build June 2021,^28^ Hospital Episodes Statistics Admitted Patient Care (HES APC), and deaths recorded by the Office of National Statistics (ONS). CPRD Aurum contains longitudinal primary care records, currently comprising >40 million individuals, and is representative of the English population’s sociodemographic profile.^29^ The dataset includes demographic and lifestyle factors, consultation records with symptoms, diagnoses, prescriptions, immunizations, tests, and referrals.^28^ Data are coded using the Systemized Nomenclature of Medicine (SNOMED), Read and local codes. HES APC contains diagnoses and procedures from National Health Service hospital inpatients in England.^30^ HES APC and ONS deaths data, containing death date and cause, are coded using International Classification of Diseases 10th version.

The CPRD Independent Scientific Advisory Committee (application 21_000428) and the London School of Hygiene and Tropical Medicine Ethics Committee (application 26191) approved the study. CPRD provided relevant linked data for the study population.

### Study design

SCCS uses within-person comparisons, i.e. individuals act as their own controls during different time periods with only individuals with the exposure (in our study influenza vaccine) and outcome (acute cardiovascular events) of interest included.^27^ SCCS analyses investigate the effect of a time-varying exposure on the outcome using conditional Poisson regression models to derive incidence ratios by comparing the incidence of events during risk time with the incidence during baseline time.^31^ As only cases are sampled, the likelihood is conditional on an event having occurred during the observation period.

The main advantage of the SCCS design is the removal of confounding due to fixed characteristics, recorded or not, that vary between individuals.^27^ In observational vaccine effectiveness studies, it is vital to remove confounding: vaccinated and unvaccinated individuals may have health, lifestyle and behavioral differences that are difficult to ascertain in routinely collected data.^32^

For the SCCS method to produce unbiased effect estimates of the association between an exposure and event, some key assumptions are required.^33,34^ First, event recurrences must be independent i.e. an event must not increase the probability of a further event. Second, an event should not impact subsequent exposure. Third, an event must not influence the end of the period of observation, but the assumption if often violated when the event increases the likelihood of mortality.

### Study population and follow-up

The source population included CPRD Aurum recorded adults aged 40–84 years with ≥12 months current post-registration time from 1 September 2009 to 31 August 2019. We ended our study in 2019 to prevent the introduction of bias due to COVID-19 circulation from the start of 2020 onwards. We identified those with a first acute cardiovascular event in the same 12-month period (1 September to 31 August) as influenza vaccination. We identified influenza vaccination records in CPRD data.^35^ In England, the influenza vaccination programme begins annually in September, ahead of the influenza season that usually occurs between December and March.^36^

We defined our outcome of any acute cardiovascular event as MI, unstable angina, acute left ventricular heart failure, stroke, transient ischaemic attack, or acute limb ischaemia. Our secondary outcomes were each of the cardiovascular conditions separated out (except for acute limb ischaemia due to small numbers). We included diagnoses coded in CPRD or HES APC.^35^ To ensure we only included first acute cardiovascular events (to meet the first SCCS assumption outlined in our study design section), we excluded individuals with a previous diagnosis (from CPRD and HES APC data), major intervention for or clinical review specific to CVD (as recorded in CPRD) before the start of follow-up. We defined CVD as heart disease (congenital or otherwise), heart failure, stroke or transient ischaemic attack.^35^

We stratified the study population by cardiovascular risk. In separate analyses, we defined cardiovascular risk by hypertension and QRISK2 score, the latter being the cardiovascular risk score used in primary care practice in England during our study period. We only included persistent and diagnosed hypertension, defined by coded CPRD diagnoses.^35^ QRISK2 uses many risk factors to estimate an individual’s absolute 10-year risk of CVD.^37^ The risk factors considered are age, sex, ethnicity, deprivation score for area of residence, family history of coronary heart disease in a first degree relative <60 years, diabetes, atrial fibrillation, chronic kidney disease Stage 4 or 5, rheumatoid arthritis, ratio of total serum cholesterol to high-density lipoprotein cholesterol, systolic blood pressure, treated hypertension, body mass index, and smoking status. We calculated QRISK2 scores using the published definitions and weights assigned to each risk factors.^37^ As part of the standard approach to calculating QRISK2 scores, we considered the absence of a code for comorbid conditions to equate to absence of the condition (i.e. if the individual had no diabetes code recorded it was determined that the individual did not have diabetes) and imputed missing lifestyle and anthropometric measures (such as body mass index) with population average values, in line with how the algorithm used by general practitioners in the calculation of QRISK2 scores works in routine clinical practice. Our full method is published online.^38^ All individuals aged ≥85 years are classed as having a QRISK2 score ≥10% due to age alone,^39^ so we limited our study population to those aged <85 years. We classified individuals as having raised cardiovascular risk (hypertension or QRISK2 score ≥10%) or not (no hypertension or QRISK2 score <10%) at baseline (1 September).

**Figure 1:**
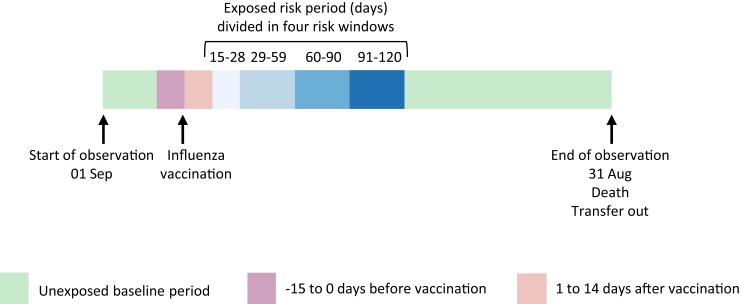
Overview of study design. Illustration of baseline and risk contributing follow-up time in relation to start of follow-up (1 September), influenza vaccine receipt and end of follow-up.

We excluded individuals who had their first acute cardiovascular event on the same day as influenza vaccination, as the two events were likely retrospectively recorded. Follow-up started on 1 September each year and ended at the earliest of; date of death, loss to follow-up (date of leaving the practice or the last data collection from the practice), or 31 August of the following year (*Figure 1*).

### Statistical analysis

We conducted all analyses in Stata (version 16). We described the baseline characteristics overall and stratified by cardiovascular risk including age group, sex, events associated with a hospital stay, events resulting in death, deaths during follow-up, and loss to follow-up.

We compared the incidence of acute cardiovascular events during risk periods following influenza vaccination with all baseline periods for each person (*Figure 1*). Our risk period was the 120 days after vaccination date, subdivided into the stratum of 15–28, 29–59, 60–90, and 91–120 days. We choose a 120-day risk window after influenza vaccination to cover the main period of influenza virus circulation. We excluded the 14 days before and the 14 days after vaccination from risk and baseline time.^21^ The 14 days before vaccination were excluded as acute cardiovascular events during this period likely affect the subsequent likelihood of receiving an influenza vaccine, a violation of a SCCS assumption (the second assumption outlined in our study design section). The 14 days after vaccination were excluded and presented separately as it can take up to 14 days for the vaccine to become effective.^40^

We calculated incidence ratios using conditional Poisson regression for acute cardiovascular events occurring within each risk period stratum compared with baseline. We adjusted for season using the binary classification of warm months (April–September) and cool months (October–March).^20^

We stratified results by age group (40–64, 65–74, and 75–84). Adults aged 40–64 years are selectively offered influenza vaccine based on specific underlying health conditions, so the individuals included in our study from this age group are not representative of the overall age group. Additional stratifying factors were sex (male and female) and the timing of vaccination (≤15 November or >15 November). Late vaccination, after mid-November, has previously been associated with reduced vaccine efficacy.^41^ Hypotheses for the difference between early and late vaccine response include an insufficient time for late recipients to develop an immune response before exposure to circulating virus, depletion of susceptibles, or differences in the characteristics and motivations for vaccination, such as late recipients being vaccinated in response to influenza epidemic levels.^42^

We performed three pre-specified sensitivity analyses. First, we repeated our initial analysis excluding fatal acute cardiovascular events. Acute cardiovascular events can result in death, violating the SCCS assumption that observation periods should end independently of event timing (the third assumption outlined in our study design section).^34^ We classified fatal events as those for which the individual’s death date was ≤30 days after the event. We also further stratified QRISK2 scores of ≥10% into 10–19% and ≥20% to consider finer definitions of cardiovascular risk.

To assess any violation of the assumption that an event should not influence subsequent exposure (the second SCCS assumption outlined in our study design section), we first assessed, using histograms, the difference in the number of days between vaccination and acute cardiovascular event by age group.^33^ We then used a sensitivity analysis to redefine our study population with follow-up from influenza vaccination date. We used a fixed follow-up until 31 August, regardless of survival, given we only had one exposure and the event, by definition, could only be after the exposure (see Supplementary material online, *Figure S1*). Therefore, all baseline time was from 121 days after vaccination until 31 August. Cardiovascular risk level was defined at the date of vaccination in this sensitivity analysis.

## Results

### Description of the study population

**Figure 2:**
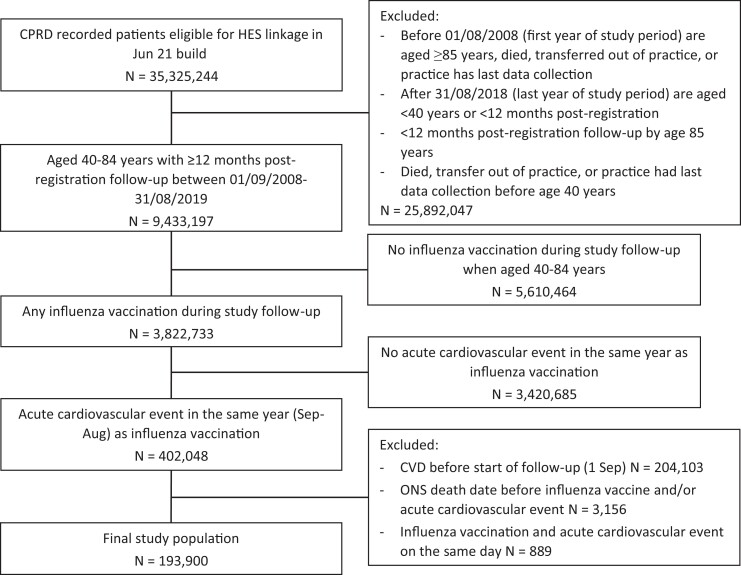
Study population flow chart. Overview of study population numbers based on inclusion and exclusion criteria. CPRD, Clinical Practice Research Datalink; CVD, cardiovascular disease; ONS, Office for National Statistics.

We included 193 900 individuals aged 40–84 years who had a first acute cardiovascular event in the same year as an influenza vaccine (*Figure 2*). 19 868 (10.2%) of individuals died and 9201 (4.7%) were lost during follow-up. Overall, 90 959 (46.9%) individuals were women, 149 663 (77.2%) were aged 65–84 years, 105 539 (54.4%) had diagnosed hypertension and 172 050 (88.7%) had a QRISK2 score of ≥10% (*Table 1*). Individuals with hypertension were older than those without hypertension [40–64 years: 17.0% (17 969) vs. 29.7% (26 268)]. Individuals with a QRISK2 score ≥10% were much older than those with a QRISK2 score <10% [40–64 years: 14.5% (24 898) vs. 88.5% (19 339)] and a higher proportion of individuals with a QRISK2 score ≥10% died compared with those with a QRISK2 score <10% [10.8% (18 641) vs. 5.6% (1227)].

**Table 1:** Baseline characteristics of the study population

	All	QRISK2		Hypertension	
		Raised risk	Low risk	Raised risk	Low risk
	*n* = 193 900	*n* = 172 050	*n* = 21 850	*n* = 105 539	*n* = 88 361
**Sex**					
Female	90 959 (46.9%)	77 453 (45.0%)	13 506 (61.8%)	52 484 (49.7%)	38 475 (43.5%)
Male	102 941 (53.1%)	94 597 (55.0%)	8344 (38.2%)	53 055 (51.3%)	49 886 (56.5%)
**Age group (years)**					
40–64	44 237 (22.8%)	24 898 (14.5%)	19 339 (88.5%)	17 969 (17.0%)	26 268 (29.7%)
65–74	68 742 (35.5%)	66 231 (38.5%)	2511 (11.5%)	36 243 (34.3%)	32 499 (36.8%)
75–84	80 921 (41.7%)	80 921 (47.0%)	0 (0.0%)	51 327 (48.6%)	29 594 (33.5%)
**Ethnicity**					
White	146 318 (75.5%)	130 207 (75.7%)	16 111 (73.7%)	79 025 (74.9%)	67 293 (76.2%)
Black	6345 (3.3%)	5645 (3.3%)	700 (3.2%)	3840 (3.6%)	2505 (2.8%)
South Asian	1206 (0.6%)	810 (0.5%)	396 (1.8%)	857 (0.8%)	349 (0.4%)
Other	9121 (4.7%)	7542 (4.4%)	1579 (7.2%)	5781 (5.5%)	3340 (3.8%)
Unknown	30 910 (15.9%)	27 846 (16.2%)	3064 (14.0%)	16 036 (15.2%)	14 874 (16.8%)
**Body mass index**					
Underweight (<18.5 kg/m^2^)	3169 (1.6%)	2781 (1.6%)	388 (1.8%)	1352 (1.3%)	1817 (2.1%)
Normal (18.5–24.9 kg/m^2^)	42 421 (21.9%)	38 053 (22.1%)	4368 (20.0%)	20 928 (19.8%)	21 493 (24.3%)
Overweight (25.0–29.9 kg/m^2^)	58 499 (30.2%)	53 005 (30.8%)	5494 (25.1%)	33 858 (32.1%)	24 641 (27.9%)
Obese (30.0–39.9 kg/m^2^)	46 425 (23.9%)	41 334 (24.0%)	5091 (23.3%)	31 208 (29.6%)	15 217 (17.2%)
Severely obese (≥40.0 kg/m^2^)	6731 (3.5%)	5665 (3.3%)	1066 (4.9%)	4871 (4.6%)	1860 (2.1%)
Unknown	36 655 (18.9%)	31 212 (18.1%)	5443 (24.9%)	13 322 (12.6%)	23 333 (26.4%)
**Smoking status**					
Current	83 692 (43.2%)	73 156 (42.5%)	10 536 (48.2%)	49 860 (47.2%)	33 832 (38.3%)
Previous	66 618 (34.4%)	61 509 (35.8%)	5109 (23.4%)	38 041 (36.0%)	28 577 (32.3%)
Never	31 521 (16.3%)	27 449 (16.0%)	4072 (18.6%)	13 735 (13.0%)	17 786 (20.1%)
Unknown	12 069 (6.2%)	9936 (5.8%)	2133 (9.8%)	3903 (3.7%)	8166 (9.2%)
Diabetes	34 257 (17.7%)	33 261 (19.3%)	996 (4.6%)	24 569 (23.3%)	9688 (11.0%)
**Cholesterol to high-density lipoprotein ratio**					
Mean (SD)	3.7 (1.2)	3.7 (1.2)	3.7 (1.2)	3.6 (1.2)	3.9 (1.3)
Unknown	59 330 (30.6%)	50 257 (29.2%)	9073 (41.5%)	23 032 (21.8%)	36 298 (41.1%)
**Associated hospital stay**					
Yes	136 426 (70.4%)	121 036 (70.3%)	15 390 (70.4%)	74 318 (70.4%)	62 108 (70.3%)
Median (IQR) stay	4.0 (2.0–10.0)	4.0 (2.0–10.0)	3.0 (1.0–7.0)	4.0 (2.0–11.0)	4.0 (2.0–9.0)
Died ≤30 days after event	13 193 (6.8%)	12 338 (7.2%)	855 (3.9%)	7604 (7.2%)	5589 (6.3%)
Died in study period	19 868 (10.2%)	18 641 (10.8%)	1227 (5.6%)	11 487 (10.9%)	8381 (9.5%)
Loss to follow-up	9201 (4.7%)	8756 (5.1%)	445 (2.0%)	5683 (5.4%)	3518 (4.0%)

### Association between influenza vaccine and first acute cardiovascular event

**Figure 3:**
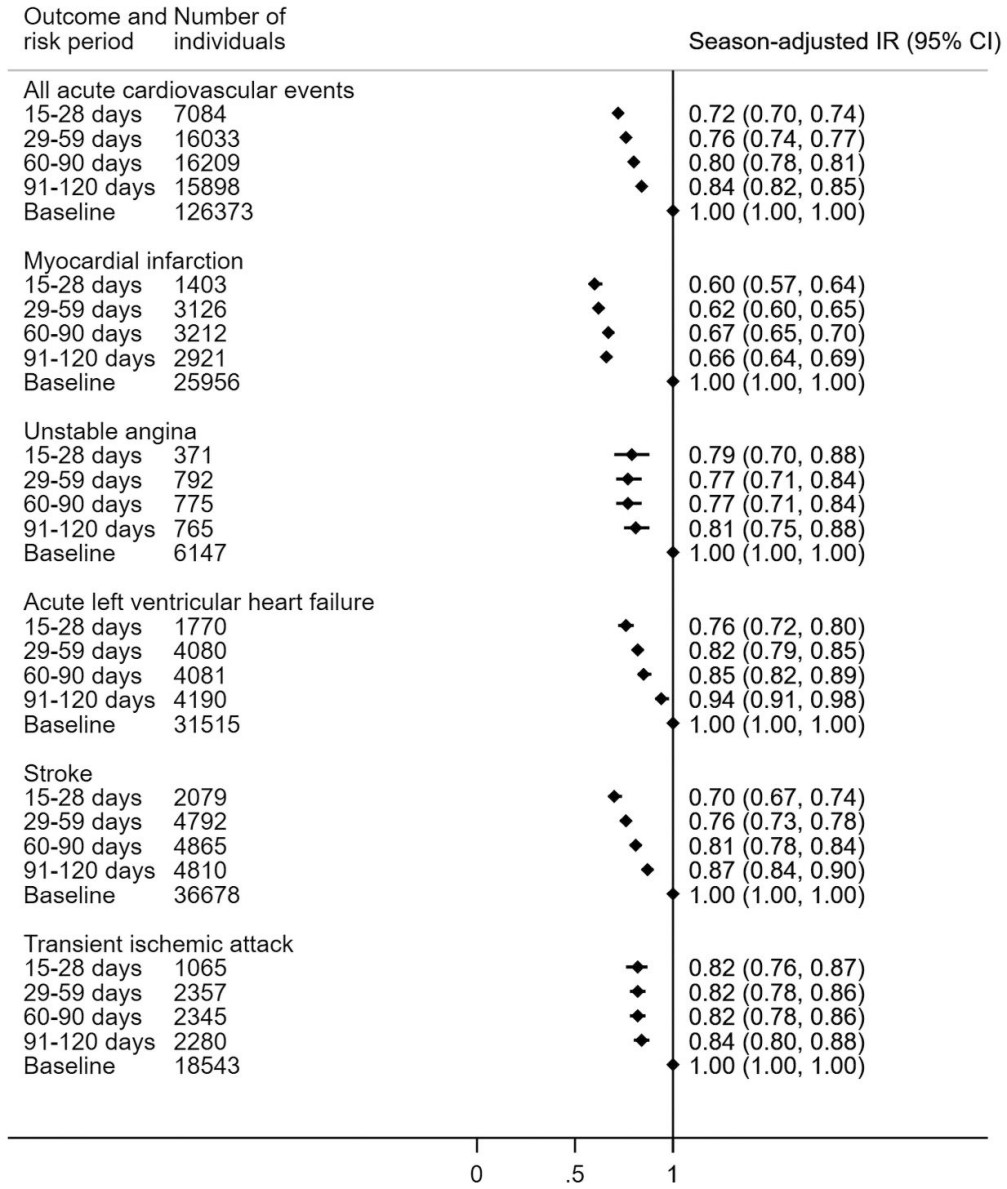
Incidence ratios for first acute cardiovascular events in risk periods following influenza vaccination by cardiovascular event type. Forest plot visualization of season-adjusted incidence ratios for primary and secondary outcomes broken down by risk periods of 15–28, 29–59, 60–90 and 91–120 days. CI, confidence interval; IR, incidence ratio.

A significant reduction in the season-adjusted incidence of first acute cardiovascular event was observed throughout the 120-day risk period after influenza vaccination. There was a tapering in the risk reduction over time; with a 28% [incidence ratio 0.72 (95% CI 0.70–0.74)] reduction 15–28 days post-vaccination and 16% [0.84 (0.82–0.85)] 91–120 days post-vaccination. When stratified by cardiovascular risk, there was a larger reduction for individuals without hypertension [15–28 days 0.66 (0.64–0.69)] than for those with hypertension [15–28 days 0.76 (0.74–0.79)]. Results were similar when raised cardiovascular risk was defined by QRISK2 score ≥10% [15–28 days 0.76 (0.74–0.78)], but there was a more substantial reduction for individuals with a QRISK2 score <10% [15–28 days 0.48 (0.44–0.52)]. The full results are in *Table 2*. Analysis of the secondary outcomes showed the reduction in incidence ratio following influenza vaccination was more substantial for MI [15–28 days 0.60 (0.57–0.64)] than other cardiovascular events (*Figure 3*). Secondary outcomes by cardiovascular risk are presented in Supplementary material online, *Figures S2–S5*.

**Table 2:** Incidence ratios for events in risk periods following influenza vaccination by cardiovascular risk and age

Risk period	All		QRISK2^a^				Hypertension			
	N events	IR (95% CI)	Raised risk		Low risk		Raised risk		Low risk	
			N events	IR (95% CI)	N events	IR (95% CI)	N events	IR (95% CI)	N events	IR (95% CI)
**All ages**										
15–28 days	7084	0.72 (0.70–0.74)	6427	0.77 (0.75–0.79)	657	0.48 (0.44–0.52)	3948	0.77 (0.75–0.80)	3136	0.67 (0.64–0.69)
29–59 days	16 033	0.76 (0.74–0.77)	14 567	0.81 (0.79–0.82)	1466	0.49 (0.46–0.52)	8906	0.81 (0.79–0.83)	7127	0.70 (0.68–0.72)
60–90 days	16 209	0.80 (0.78–0.81)	14 778	0.85 (0.84–0.87)	1431	0.50 (0.47–0.53)	9089	0.86 (0.84–0.88)	7120	0.73 (0.71–0.75)
91–120 days	15 898	0.84 (0.82–0.85)	14 465	0.89 (0.88–0.91)	1433	0.55 (0.52–0.58)	8839	0.90 (0.87–0.92)	7059	0.78 (0.76–0.80)
Baseline^b^	126 373	ref	111 291	ref	15 082	ref	68 458	ref	57 915	ref
**40–64 years** ^**c**^										
15–28 days	1402	0.54 (0.51–0.57)	837	0.62 (0.58–0.67)	565	0.45 (0.41–0.49)	592	0.63 (0.58–0.69)	810	0.49 (0.45–0.53)
29–59 days	3121	0.55 (0.53–0.58)	1845	0.63 (0.60–0.67)	1276	0.47 (0.44–0.50)	1375	0.67 (0.63–0.72)	1746	0.49 (0.46–0.51)
60–90 days	3057	0.56 (0.54–0.59)	1810	0.64 (0.61–0.68)	1247	0.48 (0.45–0.51)	1339	0.68 (0.64–0.72)	1718	0.50 (0.48–0.53)
91–120 days	3034	0.61 (0.58–0.63)	1790	0.68 (0.65–0.72)	1244	0.52 (0.49–0.56)	1340	0.72 (0.68–0.77)	1694	0.54 (0.51–0.57)
Baseline^b^	30 022	ref	16 628	ref	13 394	ref	11 970	ref	18 052	ref
**65–74 years** ^**c**^										
15–28 days	2517	0.80 (0.77–0.84)	2425	0.80 (0.77–0.84)	92	0.80 (0.64–1.00)	1346	0.82 (0.78–0.87)	1171	0.78 (0.73–0.83)
29–59 days	5699	0.84 (0.81–0.86)	5509	0.84 (0.81–0.87)	190	0.76 (0.64–0.90)	2961	0.83 (0.80–0.87)	2738	0.84 (0.80–0.88)
60–90 days	5865	0.89 (0.86–0.92)	5681	0.90 (0.87–0.92)	184	0.75 (0.63–0.89)	3107	0.90 (0.87–0.94)	2758	0.87 (0.84–0.91)
91–120 days	5769	0.93 (0.90–0.96)	5580	0.94 (0.91–0.97)	189	0.82 (0.69–0.96)	3014	0.93 (0.89–0.97)	2755	0.93 (0.89–0.97)
Baseline^b^	44 708	ref	43 020	ref	1688	ref	23 667	ref	21 041	ref
**75–84 years** ^**c**^										
15–28 days	3165	0.80 (0.77–0.83)					2010	0.80 (0.77–0.84)	1155	0.80 (0.75–0.85)
29–59 days	7213	0.86 (0.83–0.88)					4570	0.86 (0.83–0.89)	2643	0.86 (0.82–0.90)
60–90 days	7287	0.91 (0.88–0.93)					4643	0.91 (0.88–0.95)	2644	0.90 (0.86–0.94)
91–120 days	7095	0.96 (0.93–0.98)					4485	0.95 (0.92–0.99)	2610	0.96 (0.92–1.00)
Baseline^b^	51 643	ref					32 821	ref	18 822	ref

QRISK2 score results are not included for those aged 75–84 years as all individuals were high risk.

Baseline events which occurred before the vaccine risk periods were 25 730 in all ages, 11 605 in 40- to 64-year-olds, 7264 in 65- to 74-year-olds and 6861 in 75- to 84-year-olds and baseline events which occurred after the vaccine risk periods were 100 643 in all ages, 18 417 in 40- to 64-year-olds, 37 444 in 65- to 74-year-olds and 44 782 in 75- to 84-year-olds.

*P*-values for age interaction were <0.0001 in models with all age groups, i.e. with all individuals and by cardiovascular risk stratification, other than QRISK2 score <10% for which it was 0.0004.

CI, confidence interval, IR, incidence ratio.

Results were markedly different between age groups (*Table 2*) with a much larger reduction in the incidence ratio of first acute cardiovascular event in risk periods for individuals aged 40–64 years [15–28 days 0.54 (0.51–0.57)], compared with 65–74 and 75–84 years [15–28 days 0.80 (0.77–0.84) and 0.80 (0.77–0.83), respectively] (*P*-value for interaction <0.0001). The pattern was similar across all cardiovascular risk groups, although no one aged 75–84 years had a QRISK2 score <10%.

The incidence ratios stratified by sex is in Supplementary material online, *Table S1*. The reduction in incidence ratio was larger in men than women, for example incidence ratios for 15–28 days post-vaccination were 0.69 (0.67–0.72) and 0.76 (0.73–0.79), respectively (*P*-value for interaction <0.0001) compared with the baseline.

There was a slight difference in the incidence ratio of first acute cardiovascular events for individuals vaccinated on or before 15 November and after 15 November [15–28 days: 0.73 (0.71–0.75) vs. 0.69 (0.66–0.73), respectively] (*P*-value for interaction <0.0001) (see Supplementary material online, *Table S2*).

Annual breakdowns did not reveal any substantial differences in reduced incidence ratio following vaccination (see Supplementary material online, *Table S3*). Results for the first 14 days after vaccination are presented in Supplementary material online, *Table S4*.

### Sensitivity analysis removing people who had a fatal acute cardiovascular event

After exclusion of fatal acute cardiovascular events (13 193), the incidence ratios during risk periods remained broadly similar to the main analysis overall [15–28 days 0.76 (0.74–0.78) and 91–120 days 0.81 (0.79–0.82 )] and across all cardiovascular risk groups (see Supplementary material online, *Table S5*).

### Sensitivity analysis with more refined QRISK2 score stratification

When raised cardiovascular risk defined by QRISK2 score was separated into 10–19% and ≥20%, the reduction in incidence ratio during risk periods, compared with the baseline, among individuals aged 65–74 years was greater in those with a risk score ≥20% [15–28 days 0.79 (0.74–0.83)] than a risk score of 10–19% [0.82 (0.77–0.88)] but still broadly similar. Most individuals aged 75–84 years had a QRISK2 score ≥20%. Among those aged 40–64 years, the reduction was greater in those with a QRISK2 score of 10–19% (see Supplementary material online, *Table S6*).

### Sensitivity analysis study design

Our investigation of the timing of the event centred to vaccination showed that a high number of events in individuals aged 40–64 years occurred prior to vaccination (see Supplementary material online, *Figure S6*). Overall the baseline characteristics of the sensitivity analysis study population were similar to those of the main study population (see Supplementary material online, *Table S7*), but they were slightly older [40–64 years: 18.6% (29 927) vs. 22.8% (44 237)] with a higher proportion of individuals having a QRISK2 score of ≥10% [91.4% (147 023) vs. 88.7% (172 050)]. Compared with the main study design and population, there was a smaller incidence ratio reduction of a first acute cardiovascular event during early risk periods after vaccination [15–28 days 0.94 (0.91–0.96)] and no reduction by 91–120 days [1.00 (0.98–1.02)] (see Supplementary material online, *Table S8*). Among individuals aged 40–64 years there was no difference in incidence ratio during risk periods compared with the baseline (see Supplementary material online, *Table S8*).

## Discussion

### Summary

Using English primary and secondary data electronic health records from 2008 to 2019, we found individuals with both raised and low cardiovascular risk had a reduced incidence of a first acute cardiovascular event after influenza vaccination after adjusting for season [we used a binary classification of warmer and cooler months but when season was adjusted for using four season (results not shown) the change in association was the same]. The reduced incidence was largest in the 15–28 days after vaccination but persisted to 120 days. The effect size varied from 6 to 28% across different analyses of study population groups. The protective effect was evident across all age groups in the main analyses but was confined to those ≥65 years in the final sensitivity analysis with follow-up from vaccination date (*Structured Graphical Abstract*).

### Comparison with existing literature

Our main finding for the whole study population was consistent with the results generated by previous SCCS studies using CPRD data. Analysis of data from 1987 to 2001 found a 12% [IR 0.88 ( 0.80–0.97)] and 13% [IR 0.87 (0.79–0.96)] reduction in relative incidence of first stroke and MI, respectively, in the 15–28 days after influenza vaccination after which time there was no significant reduction.^20^ Two other SCCS studies with CPRD data from 2001 to 2009 found incidence ratios of 0.75 (0.66–0.86) and 0.76 (0.70–0.84) for first MI and stroke, respectively, in the 15–28 days post-vaccination.^21,22^ Although we used a composite acute cardiovascular event outcome, when we looked at individual cardiovascular outcomes, the greatest reduction in relative incidence was for MI.

Previous studies have shown that individuals with raised cardiovascular risk have more acute cardiovascular complications following respiratory infection.^25,26^ Sen *et al.* used Norwegian electronic health record data from 2009 to 2010 to investigate the impact of underlying cardiovascular risk on the association between the H1N1 influenza vaccine and cardiovascular events.^23^ The study identified conflicting results, with a reduction in the incidence ratio of MI [15–28 days post-vaccination: 0.70 (0.57–0.85)] in those with raised cardiovascular risk and an increase [15–28 days post-vaccination: 3.17 (1.99–5.07)] among people at low cardiovascular risk. The study defined cardiovascular risk using cardiovascular prevention prescriptions at the time of vaccination, after follow-up had started, which likely biased results when stratified by cardiovascular risk so comparisons to our results is difficult.

We showed similar protective associations between influenza vaccination and acute cardiovascular events regardless of cardiovascular risk level in people aged ≥65 years. However, among those aged 40–64 years, there was an apparently greater protective association in those at low underlying cardiovascular risk in main analysis (though not in our sensitivity analysis study design). Individuals who have low cardiovascular risk or who are younger have a lower baseline risk of cardiovascular complications, whereas individuals with raised cardiovascular risk or who are older have a high risk all year round. In higher risk older people influenza-associated cardiovascular complications may explain a lower proportion of cardiovascular events.

### Strengths and limitations

We used a large study population from primary and secondary care linked data sources generalizable to the English population. The large study population allowed us to stratify simultaneously by cardiovascular risk, age, and a third factor such as sex. Thereby, allowing us to unpick the findings of our initial analysis in more detail than previous SCCS studies. We compared results across two measures of cardiovascular risk; QRISK2 score and diagnosed hypertension. As our study population was predominantly older, most individuals had a QRISK2 score ≥10%, limiting our ability to conclude any added benefit influenza vaccine may have in younger people with raised cardiovascular risk.

Observational studies, particularly those involving secondary analysis of routinely collected data, of vaccine effects are highly vulnerable to confounding as vaccinated individuals tend to have health, lifestyle, and behavioral differences to those who are not vaccinated.^32^ The SCCS design largely overcomes confounding by such fixed individual characteristic by using within-individual comparisons. The design does not control for time-varying confounders within individuals. However, we believe time-varying confounding is likely to be minimized in our study due to the maximum 1-year follow-up, with adjustment for season. Another bias not controlled for in the SCCS design is healthcare contact bias. It is possible that healthcare contact for influenza vaccination could lead to the initiation of other cardiovascular prevention strategies which might reduce the incidence of subsequent cardiovascular events in time periods immediately following vaccination. However, as many primary care practices in England run specific influenza vaccination clinics, this is unlikely to have majorly impacted results. While we removed Days −14 to −1 from baseline time to avoid violation of the SCCS assumption that the occurrence of an outcome should not affect the probability of exposure, it is also possible that early symptoms of myocardial ischaemia might prevent an individual from attending for influenza vaccine leading to low relative incidence of acute cardiovascular events in the earliest time-period after vaccination. While it is not possible for such bias to be quantified, it is most likely to affect the results of Days 1–7, while protection demonstrated in Days 8–14 could plausibly be due to a swift antibody response followed by later antibody waning.^43,44^ Further research could utilize a negative outcome control, though would need to carefully select a suitable acute event which would not be associated with influenza vaccine. The representativeness of the individuals included aged <65 years and ≥65 years differs. In England before the COVID-19 pandemic, universal influenza vaccination only included individuals aged ≥65 years. Before this age, the influenza vaccine was only offered (free of charge) to those with an underlying health condition. One trigger for offering influenza vaccination would be a recent acute cardiovascular event. A high proportion of events in individuals aged <65 years (26%) occurred before vaccination, compared with low proportions in those aged 65–74 years (11%) and 75–84 years (8%). This difference suggests that events led to vaccination in some individuals aged <65 years and may explain why our sensitivity analysis study design which began follow-up at vaccination, showed no protective association in those aged <65 years. Conversely, a higher proportion of older individuals died after their event, which resulted in a short baseline interval in our main study design again potential causing bias, although results from analysis of only non-fatal events suggest this bias was small.

CPRD and HES are widely validated,^45^ including for cardiovascular events such as MI, heart failure and stroke.^46^ Influenza vaccine recording in CPRD has not been specifically validated. Most patients in England will receive their influenza vaccine at their primary care practice, but some vaccines will be administrated by pharmacies or occupational health services. Vaccines received outside of primary care practice are still expected to be recorded within primary care records.^47^ Both the overall influenza vaccine uptake and the patterns of regional variation are consistent with national surveillance.^48^

### Clinical and public health implications

Measuring the burden and impact of seasonal influenza is difficult, but World Health Organization estimates before the COVID-19 pandemic suggested that influenza infected approximately 20% of people in Europe, depending on the circulating strains.^49^ This poses a significant winter healthcare pressure and associated mortality of tens of thousands of deaths in Europe, with an estimated 400 000 respiratory deaths globally.^50^ In the United States, during the 2018–2019 influenza season there was an estimated 380 000 respiratory hospitalizations and 28 000 respiratory deaths.^51^ Further evidence suggests that influenza vaccine among individuals with CVD reduces mortality risk by more than one-third.^52^

The mechanisms by which influenza vaccine exerts cardiovascular benefit are uncertain. Here, we have assumed the protective effect is due to prevention of influenza which can trigger a cardiovascular event. However, there is also the possibility of pleiotropic effects between virus and the antigens of atherosclerotic plaque as well as unspecific immunomodulatory effect which in turn prevents cardiovascular complications unrelated to influenza virus circulation and infection.^53^ Consideration of the different mechanistic and long-term effects should be explored in future research.

Among adults aged <65 years, some with high cardiovascular risk are already eligible to receive influenza vaccine in many European countries, including those with chronic kidney disease, severe obesity, or diabetes. However, uptake among clinical risk groups is currently moderate in England and low in other European countries.^8,9^ A SCCS investigating the association between influenza vaccination and hospitalization risk in heart failure patients identified uptake of vaccination among the study population was low but associated with a lower risk of hospitalization due to CVD after vaccination.^54^ Low uptake may be due to individual or physician perceived risk. Age-eligible vaccination is operationally easier to manage. During the COVID-19 pandemic, influenza vaccine recommendations in England have been extended to all individuals ≥50 years regardless of underlying health conditions.^9^ While further studies would help to fully characterize those who would derive the most cardiovascular benefit from influenza vaccine, improving uptake remains a public health priority, both to protect individuals from influenza and complications, including cardiovascular events. On summarizing the evidence generated from RCTs and observations studies to date, a recent editorial emphasized the need for cardiologists, and other physicians, to consider the cardiovascular benefits of influenza vaccine and ensure their patients receive the vaccine in the same way they would advocate the use of statins.^19^ Such promotion of influenza vaccine would be a step towards a ‘syndemic’ approach to healthcare, acknowledging the interaction of infectious diseases and non-communicable diseases, such as CVD.^55^ Ultimately, in-hospital vaccination of those hospitalized due to, or at high risk of, cardiovascular complication is likely one of the most efficient ways to increase vaccine uptake.

## Conclusions

We have shown that influenza vaccine is associated with reduced risk of cardiovascular events, regardless of underlying cardiovascular risk. Improved vaccine uptake could help reduce the risk of first acute cardiovascular events among those already eligible to receive the seasonal influenza vaccine. Furthermore, with continued widespread COVID-19 transmission, minimizing influenza impact is crucial. COVID-19 vaccine boosters are currently being rolled out and offer the opportunity to increase and prioritize influenza vaccine uptake.^56^

## Supplementary Material

ehac737_Supplementary_Data

## Data Availability

The data used for this study were obtained from the CPRD. All CPRD data are available via an application to the Independent Scientific Advisory Committee (see https://www.cprd.com/Data-access). Data acquisition is associated with a fee and data protection requirements. This study is supported by code lists used to define each health condition, which have been made openly available at https://doi.org/10.17037/DATA.00002675. Our data preparation and analysis Stata code is available via GitHub at https://github.com/jenAdavidson/sccs_fluvacc_cvd. All code is shared without investigator support.
