# Supplementary material for: Primary prevention of acute cardiovascular events by influenza vaccination: an observational study
Source: Eur Heart J. 2022 Dec 20;44(7):610–20. doi: 10.1093/eurheartj/ehac737 (PMC9925273; doi:10.1093/eurheartj/ehac737)
Supplement: ehac737_Supplementary_Data [file ehac737_supplementary_data.docx]

Supplementary Table 1. Incidence ratios for first acute cardiovascular event in risk periods following influenza vaccination by cardiovascular risk, age group and sex

| Risk period | All | | QRISK2 | | | | Hypertension | | | |
| --- | --- | --- | --- | --- | --- | --- | --- | --- | --- | --- |
|  |  |  | Raised risk | | Low risk | | Raised risk | | Low risk | |
|  | N events | IR (95% CI) | N events | IR (95% CI) | N events | IR (95% CI) | N events | IR (95% CI) | N events | IR (95% CI) |
| All ages |  | p <0.0001 |  | p <0.0001 |  | p <0.0001 |  | p = 0.0003 |  | p <0.0001 |
| Women |  |  |  |  |  |  |  |  |  |  |
| 15-28 days | 3,377 | 0.76 (0.73-0.79) | 2,909 | 0.79 (0.76-0.82) | 468 | 0.62 (0.56-0.68) | 1,986 | 0.78 (0.75-0.82) | 1,391 | 0.72 (0.68-0.77) |
| 29-59 days | 7,699 | 0.80 (0.78-0.82) | 6,709 | 0.85 (0.82-0.87) | 990 | 0.60 (0.56-0.65) | 4,500 | 0.82 (0.79-0.85) | 3,199 | 0.77 (0.74-0.81) |
| 60-90 days | 7,773 | 0.84 (0.82-0.86) | 6,762 | 0.89 (0.86-0.92) | 1,011 | 0.64 (0.60-0.69) | 4,494 | 0.86 (0.83-0.89) | 3,279 | 0.82 (0.79-0.86) |
| 91-120 days | 7,519 | 0.87 (0.85-0.90) | 6,554 | 0.93 (0.90-0.95) | 965 | 0.66 (0.61-0.70) | 4,345 | 0.89 (0.86-0.92) | 3,174 | 0.85 (0.82-0.89) |
| Baseline | 59,150 | ref | 50,051 | ref | 9,099 | ref | 34,098 | ref | 25,052 | ref |
| Men |  |  |  |  |  |  |  |  |  |  |
| 15-28 days | 3,707 | 0.69 (0.67-0.72) | 3,518 | 0.75 (0.72-0.78) | 189 | 0.31 (0.27-0.36) | 1,962 | 0.76 (0.73-0.80) | 1,745 | 0.63 (0.60-0.66) |
| 29-59 days | 8,334 | 0.72 (0.70-0.74) | 7,858 | 0.78 (0.76-0.80) | 476 | 0.36 (0.33-0.40) | 4,406 | 0.79 (0.77-0.82) | 3,928 | 0.66 (0.63-0.68) |
| 60-90 days | 8,436 | 0.76 (0.74-0.78) | 8,016 | 0.82 (0.80-0.85) | 420 | 0.34 (0.30-0.37) | 4,595 | 0.86 (0.83-0.89) | 3,841 | 0.67 (0.64-0.69) |
| 91-120 days | 8,379 | 0.81 (0.79-0.83) | 7,911 | 0.87 (0.85-0.89) | 468 | 0.42 (0.38-0.46) | 4,494 | 0.90 (0.87-0.93) | 3,885 | 0.73 (0.70-0.76) |
| Baseline | 67,223 | ref | 61,240 | ref | 5,983 | ref | 34,360 | ref | 32,863 | ref |
| 40-64 years |  | p <0.0001 |  | p <0.0001 |  | p <0.0001 |  | p = 0.0021 |  | p <0.0001 |
| Women |  |  |  |  |  |  |  |  |  |  |
| 15-28 days | 597 | 0.64 (0.59-0.70) | 220 | 0.74 (0.64-0.86) | 377 | 0.59 (0.53-0.66) | 258 | 0.72 (0.63-0.82) | 339 | 0.59 (0.52-0.66) |
| 29-59 days | 1,337 | 0.66 (0.62-0.70) | 532 | 0.83 (0.75-0.91) | 805 | 0.58 (0.54-0.63) | 589 | 0.75 (0.68-0.83) | 748 | 0.60 (0.55-0.65) |
| 60-90 days | 1,350 | 0.69 (0.65-0.73) | 522 | 0.83 (0.75-0.92) | 828 | 0.62 (0.58-0.67) | 559 | 0.73 (0.67-0.81) | 791 | 0.66 (0.61-0.71) |
| 91-120 days | 1,262 | 0.69 (0.65-0.73) | 483 | 0.82 (0.74-0.91) | 779 | 0.63 (0.58-0.68) | 519 | 0.73 (0.66-0.80) | 743 | 0.67 (0.62-0.72) |
| Baseline | 11,639 | ref | 4,207 | ref | 7,432 | ref | 4,725 | ref | 6,914 | ref |
| Men |  |  |  |  |  |  |  |  |  |  |
| 15-28 days | 805 | 0.49 (0.45-0.52) | 617 | 0.59 (0.54-0.64) | 188 | 0.31 (0.27-0.36) | 334 | 0.58 (0.52-0.65) | 471 | 0.44 (0.40-0.48) |
| 29-59 days | 1,784 | 0.50 (0.47-0.52) | 1,313 | 0.58 (0.55-0.62) | 471 | 0.36 (0.32-0.40) | 786 | 0.63 (0.58-0.68) | 998 | 0.43 (0.40-0.46) |
| 60-90 days | 1,707 | 0.50 (0.47-0.52) | 1,288 | 0.59 (0.55-0.63) | 419 | 0.34 (0.30-0.37) | 780 | 0.64 (0.59-0.69) | 927 | 0.42 (0.39-0.45) |
| 91-120 days | 1,772 | 0.56 (0.53-0.59) | 1,307 | 0.65 (0.61-0.69) | 465 | 0.42 (0.38-0.46) | 821 | 0.72 (0.67-0.78) | 951 | 0.47 (0.44-0.51) |
| Baseline | 18,383 | ref | 12,421 | ref | 5,962 | ref | 7,245 | ref | 11,138 | ref |
| 65-74 years |  | p = 0.0176 |  | p = 0.0308 |  | p = 0.2686 |  | p = 0.0022 |  | p = 0.5397 |
| Women |  |  |  |  |  |  |  |  |  |  |
| 15-28 days | 1,143 | 0.82 (0.77-0.87) | 1,052 | 0.82 (0.77-0.88) | 91 | 0.80 (0.64-1.00) | 642 | 0.84 (0.77-0.92) | 501 | 0.79 (0.72-0.87) |
| 29-59 days | 2,491 | 0.82 (0.79-0.86) | 2,306 | 0.83 (0.79-0.87) | 185 | 0.75 (0.63-0.89) | 1,310 | 0.79 (0.74-0.84) | 1,181 | 0.86 (0.81-0.93) |
| 60-90 days | 2,534 | 0.86 (0.82-0.91) | 2,351 | 0.87 (0.83-0.92) | 183 | 0.76 (0.64-0.90) | 1,341 | 0.83 (0.78-0.89) | 1,193 | 0.90 (0.84-0.96) |
| 91-120 days | 2,479 | 0.90 (0.86-0.94) | 2,293 | 0.91 (0.87-0.95) | 186 | 0.81 (0.69-0.96) | 1,335 | 0.89 (0.83-0.95) | 1,144 | 0.92 (0.85-0.98) |
| Baseline | 20,010 | ref | 18,343 | ref | 1,667 | ref | 10,959 | ref | 9,051 | ref |
| Men |  |  |  |  |  |  |  |  |  |  |
| 15-28 days | 1,374 | 0.79 (0.74-0.84) | 1,373 | 0.79 (0.74-0.84) | 1 | 0.70 (0.08-5.97) | 704 | 0.81 (0.74-0.88) | 670 | 0.77 (0.71-0.84) |
| 29-59 days | 3,208 | 0.85 (0.81-0.89) | 3,203 | 0.85 (0.81-0.89) | 5 | 1.63 (0.48-5.56) | 1,651 | 0.87 (0.82-0.93) | 1,557 | 0.83 (0.78-0.88) |
| 60-90 days | 3,331 | 0.91 (0.87-0.95) | 3,330 | 0.91 (0.87-0.95) | 1 | 0.36 (0.04-3.07) | 1,766 | 0.97 (0.91-1.02) | 1,565 | 0.86 (0.81-0.91) |
| 91-120 days | 3,290 | 0.96 (0.92-1.00) | 3,287 | 0.96 (0.92-1.00) | 3 | 1.13 (0.28-4.63) | 1,679 | 0.97 (0.92-1.03) | 1,611 | 0.94 (0.89-1.00) |
| Baseline | 24,698 | ref | 24,677 | ref | 21 | ref | 12,708 | ref | 11,990 | ref |
| 75-84 years |  | p = 0.2316 |  |  |  |  |  | p = 0.1814 |  | p = 0.8404 |
| Women |  |  |  |  |  |  |  |  |  |  |
| 15-28 days | 1,637 | 0.78 (0.74-0.83) |  |  |  |  | 1,086 | 0.77 (0.73-0.83) | 551 | 0.80 (0.73-0.88) |
| 29-59 days | 3,871 | 0.86 (0.83-0.90) |  |  |  |  | 2,601 | 0.87 (0.83-0.91) | 1,270 | 0.86 (0.80-0.92) |
| 60-90 days | 3,889 | 0.91 (0.88-0.95) |  |  |  |  | 2,594 | 0.91 (0.87-0.95) | 1,295 | 0.92 (0.86-0.98) |
| 91-120 days | 3,778 | 0.96 (0.92-1.00) |  |  |  |  | 2,491 | 0.95 (0.90-0.99) | 1,287 | 0.98 (0.92-1.05) |
| Baseline | 27,501 | ref |  |  |  |  | 18,414 | ref | 9,087 | ref |
| Men |  |  |  |  |  |  |  |  |  |  |
| 15-28 days | 1,528 | 0.83 (0.78-0.87) |  |  |  |  | 924 | 0.84 (0.78-0.91) | 604 | 0.80 (0.73-0.87) |
| 29-59 days | 3,342 | 0.85 (0.81-0.88) |  |  |  |  | 1,969 | 0.84 (0.80-0.89) | 1,373 | 0.85 (0.80-0.91) |
| 60-90 days | 3,398 | 0.90 (0.87-0.94) |  |  |  |  | 2,049 | 0.92 (0.87-0.97) | 1,349 | 0.88 (0.83-0.94) |
| 91-120 days | 3,317 | 0.95 (0.92-0.99) |  |  |  |  | 1,994 | 0.97 (0.92-1.02) | 1,323 | 0.94 (0.88-1.00) |
| Baseline | 24,142 | ref |  |  |  |  | 14,407 | ref | 9,735 | ref |

QRISK2 score results are not included for those aged 75-84 years as all individuals were high risk. P-values in table are for sex interaction

Supplementary Table 2. Incidence ratios for first acute cardiovascular event in risk periods following influenza vaccination by cardiovascular risk, age group and vaccination timing

| Risk period | All | | QRISK2 | | | | Hypertension | | | |
| --- | --- | --- | --- | --- | --- | --- | --- | --- | --- | --- |
|  |  |  | Raised risk | | Low risk | | Raised risk | | Low risk | |
|  | N events | IR (95% CI) | N events | IR (95% CI) | N events | IR (95% CI) | N events | IR (95% CI) | N events | IR (95% CI) |
| All ages |  | p <0.0001 |  | p <0.0001 |  | p <0.0001 |  | p <0.0001 |  | p = 0.0004 |
| Vaccinated on or before 15 November | | |  |  |  |  |  |  |  |  |
| 15-28 days | 5,291 | 0.73 (0.71-0.75) | 4,833 | 0.76 (0.73-0.78) | 458 | 0.57 (0.51-0.62) | 2,990 | 0.76 (0.73-0.79) | 2,301 | 0.70 (0.67-0.73) |
| 29-59 days | 12,046 | 0.77 (0.76-0.79) | 11,038 | 0.80 (0.79-0.82) | 1,008 | 0.58 (0.54-0.62) | 6,830 | 0.81 (0.78-0.83) | 5,216 | 0.73 (0.71-0.76) |
| 60-90 days | 12,303 | 0.82 (0.80-0.84) | 11,288 | 0.86 (0.84-0.87) | 1,015 | 0.61 (0.57-0.65) | 7,023 | 0.86 (0.84-0.89) | 5,280 | 0.77 (0.75-0.80) |
| 91-120 days | 12,098 | 0.87 (0.85-0.88) | 11,113 | 0.90 (0.88-0.92) | 985 | 0.63 (0.59-0.68) | 6,834 | 0.90 (0.88-0.93) | 5,264 | 0.83 (0.80-0.85) |
| Baseline | 94,592 | ref | 84,764 | ref | 9,828 | ref | 52,666 | ref | 41,926 | ref |
| Vaccinated after 15 November | | |  |  |  |  |  |  |  |  |
| 15-28 days | 1,793 | 0.69 (0.66-0.73) | 1,594 | 0.80 (0.76-0.84) | 199 | 0.33 (0.29-0.38) | 958 | 0.81 (0.76-0.87) | 835 | 0.59 (0.55-0.63) |
| 29-59 days | 3,987 | 0.71 (0.68-0.73) | 3,529 | 0.82 (0.79-0.85) | 458 | 0.35 (0.31-0.38) | 2,076 | 0.82 (0.78-0.86) | 1,911 | 0.62 (0.59-0.65) |
| 60-90 days | 3,906 | 0.72 (0.69-0.74) | 3,490 | 0.84 (0.81-0.87) | 416 | 0.32 (0.29-0.36) | 2,066 | 0.84 (0.80-0.89) | 1,840 | 0.61 (0.58-0.65) |
| 91-120 days | 3,800 | 0.76 (0.73-0.78) | 3,352 | 0.87 (0.83-0.90) | 448 | 0.39 (0.35-0.43) | 2,005 | 0.88 (0.84-0.93) | 1,795 | 0.65 (0.62-0.69) |
| Baseline | 31,781 | ref | 26,527 | ref | 5,254 | ref | 15,792 | ref | 15,989 | ref |
| 40-64 years |  | p <0.0001 |  | p = 0.0053 |  | p <0.0001 |  | p = 0.0186 |  | p <0.0001 |
| Vaccinated on or before 15 November | | |  |  |  |  |  |  |  |  |
| 15-28 days | 961 | 0.61 (0.57-0.66) | 572 | 0.68 (0.62-0.74) | 389 | 0.54 (0.49-0.60) | 420 | 0.70 (0.63-0.78) | 541 | 0.56 (0.51-0.62) |
| 29-59 days | 2,138 | 0.63 (0.60-0.66) | 1,275 | 0.70 (0.66-0.74) | 863 | 0.55 (0.51-0.60) | 960 | 0.74 (0.68-0.79) | 1,178 | 0.57 (0.53-0.60) |
| 60-90 days | 2,143 | 0.66 (0.63-0.69) | 1,268 | 0.72 (0.67-0.76) | 875 | 0.59 (0.55-0.64) | 962 | 0.76 (0.71-0.82) | 1,181 | 0.60 (0.56-0.64) |
| 91-120 days | 2,115 | 0.70 (0.66-0.73) | 1,279 | 0.77 (0.73-0.82) | 836 | 0.61 (0.57-0.66) | 962 | 0.81 (0.75-0.87) | 1,153 | 0.63 (0.59-0.67) |
| Baseline | 19,828 | ref | 11,234 | ref | 8,594 | ref | 8,201 | ref | 11,627 | ref |
| Vaccinated after 15 November | | |  |  |  |  |  |  |  |  |
| 15-28 days | 441 | 0.40 (0.36-0.44) | 265 | 0.50 (0.44-0.57) | 176 | 0.31 (0.27-0.36) | 172 | 0.48 (0.41-0.57) | 269 | 0.36 (0.32-0.41) |
| 29-59 days | 983 | 0.41 (0.38-0.44) | 570 | 0.49 (0.45-0.54) | 413 | 0.33 (0.30-0.37) | 415 | 0.53 (0.48-0.60) | 568 | 0.35 (0.32-0.38) |
| 60-90 days | 914 | 0.39 (0.36-0.42) | 542 | 0.48 (0.43-0.53) | 372 | 0.30 (0.27-0.34) | 377 | 0.49 (0.44-0.55) | 537 | 0.34 (0.31-0.37) |
| 91-120 days | 919 | 0.43 (0.40-0.47) | 511 | 0.50 (0.45-0.55) | 408 | 0.37 (0.34-0.42) | 378 | 0.54 (0.49-0.61) | 541 | 0.38 (0.35-0.42) |
| Baseline | 10,194 | Ref | 5,394 | ref | 4,800 | ref | 3,769 | ref | 6,425 | ref |
| 65-74 years |  | p = 0.1323 |  | p = 0.0561 |  | p = 0.5096 |  | p = 0.0043 |  | p = 0.2365 |
| Vaccinated on or before 15 November | | |  |  |  |  |  |  |  |  |
| 15-28 days | 1,884 | 0.79 (0.75-0.83) | 1,815 | 0.79 (0.75-0.83) | 69 | 0.84 (0.65-1.08) | 1,003 | 0.79 (0.74-0.85) | 881 | 0.79 (0.73-0.85) |
| 29-59 days | 4,297 | 0.83 (0.80-0.86) | 4,152 | 0.83 (0.80-0.86) | 145 | 0.81 (0.67-0.98) | 2,309 | 0.84 (0.80-0.88) | 1,988 | 0.82 (0.78-0.87) |
| 60-90 days | 4,419 | 0.88 (0.85-0.92) | 4,279 | 0.89 (0.86-0.92) | 140 | 0.80 (0.66-0.97) | 2,360 | 0.89 (0.85-0.93) | 2,059 | 0.88 (0.84-0.93) |
| 91-120 days | 4,398 | 0.94 (0.90-0.97) | 4,249 | 0.94 (0.91-0.97) | 149 | 0.89 (0.74-1.08) | 2,313 | 0.93 (0.88-0.97) | 2,085 | 0.95 (0.90-1.00) |
| Baseline | 33,871 | ref | 32,637 | ref | 1,234 | ref | 18,273 | ref | 15,598 | ref |
| Vaccinated after 15 November | | |  |  |  |  |  |  |  |  |
| 15-28 days | 633 | 0.84 (0.77-0.91) | 610 | 0.85 (0.77-0.92) | 23 | 0.69 (0.44-1.08) | 343 | 0.94 (0.83-1.06) | 290 | 0.75 (0.66-0.85) |
| 29-59 days | 1,402 | 0.86 (0.80-0.91) | 1,357 | 0.87 (0.81-0.93) | 45 | 0.62 (0.44-0.88) | 652 | 0.82 (0.75-0.90) | 750 | 0.89 (0.81-0.97) |
| 60-90 days | 1,446 | 0.91 (0.85-0.97) | 1,402 | 0.92 (0.86-0.98) | 44 | 0.62 (0.44-0.87) | 747 | 0.97 (0.89-1.06) | 699 | 0.85 (0.77-0.93) |
| 91-120 days | 1,371 | 0.92 (0.86-0.98) | 1,331 | 0.93 (0.87-0.99) | 40 | 0.60 (0.42-0.86) | 701 | 0.97 (0.88-1.06) | 670 | 0.87 (0.79-0.95) |
| Baseline | 10,837 | ref | 10,383 | ref | 454 | ref | 5,394 | ref | 5,443 | ref |
| 75-84 years |  | p <0.0001 |  |  |  |  |  | p <0.0001 |  | p = 0.0426 |
| Vaccinated on or before 15 November | | |  |  |  |  |  |  |  |  |
| 15-28 days | 2,446 | 0.76 (0.73-0.80) |  |  |  |  | 1,567 | 0.77 (0.73-0.81) | 879 | 0.76 (0.70-0.81) |
| 29-59 days | 5,611 | 0.82 (0.79-0.84) |  |  |  |  | 3,561 | 0.81 (0.78-0.85) | 2,050 | 0.83 (0.78-0.87) |
| 60-90 days | 5,741 | 0.88 (0.85-0.91) |  |  |  |  | 3,701 | 0.89 (0.85-0.92) | 2,040 | 0.86 (0.82-0.91) |
| 91-120 days | 5,585 | 0.93 (0.90-0.96) |  |  |  |  | 3,559 | 0.93 (0.89-0.96) | 2,026 | 0.93 (0.88-0.98) |
| Baseline | 40,893 | ref |  |  |  |  | 26,192 | ref | 14,701 | ref |
| Vaccinated after 15 November | | |  |  |  |  |  |  |  |  |
| 15-28 days | 719 | 0.98 (0.90-1.06) |  |  |  |  | 443 | 0.98 (0.88-1.09) | 276 | 0.98 (0.86-1.12) |
| 29-59 days | 1,602 | 1.02 (0.96-1.09) |  |  |  |  | 1,009 | 1.05 (0.97-1.13) | 593 | 0.98 (0.89-1.09) |
| 60-90 days | 1,546 | 1.03 (0.97-1.10) |  |  |  |  | 942 | 1.03 (0.95-1.11) | 604 | 1.05 (0.95-1.16) |
| 91-120 days | 1,510 | 1.09 (1.03-1.16) |  |  |  |  | 926 | 1.09 (1.01-1.18) | 584 | 1.09 (0.99-1.21) |
| Baseline | 10,750 | ref |  |  |  |  | 6,629 | ref | 4,121 | ref |

QRISK2 score results are not included for those aged 75-84 years as all individuals were high risk. P-values in table are for timing interactionSupplementary Table 3. Incidence ratios for first acute cardiovascular event in risk periods following influenza vaccination by year

| Risk period | 2008/09 | | 2009/10 | | 2010/11 | | 2011/12 | |
| --- | --- | --- | --- | --- | --- | --- | --- | --- |
|  | N events | IR (95% CI) | N events | IR (95% CI) | N events | IR (95% CI) | N events | IR (95% CI) |
| 15-28 days | 641 | 0.79 (0.72-0.86) | 605 | 0.72 (0.66-0.78) | 631 | 0.76 (0.69-0.82) | 586 | 0.71 (0.65-0.78) |
| 29-59 days | 1,313 | 0.76 (0.71-0.81) | 1,383 | 0.76 (0.71-0.81) | 1,320 | 0.74 (0.69-0.79) | 1,332 | 0.75 (0.70-0.80) |
| 60-90 days | 1,489 | 0.90 (0.84-0.95) | 1,367 | 0.79 (0.74-0.84) | 1,377 | 0.80 (0.75-0.86) | 1,360 | 0.80 (0.75-0.85) |
| 91-120 days | 1,375 | 0.89 (0.84-0.95) | 1,281 | 0.79 (0.75-0.85) | 1,284 | 0.81 (0.76-0.86) | 1,360 | 0.85 (0.80-0.91) |
| Baseline | 10,095 | ref | 10,611 | ref | 10,461 | ref | 10,675 | ref |
| Risk period | 2012/13 | | 2013/14 | | 2014/15 | | 2015/16 | |
|  | N events | IR (95% CI) | N events | IR (95% CI) | N events | IR (95% CI) | N events | IR (95% CI) |
| 15-28 days | 604 | 0.69 (0.64-0.76) | 622 | 0.71 (0.65-0.78) | 635 | 0.71 (0.66-0.78) | 667 | 0.74 (0.68-0.81) |
| 29-59 days | 1,484 | 0.79 (0.75-0.84) | 1,389 | 0.74 (0.69-0.78) | 1,512 | 0.79 (0.74-0.84) | 1,486 | 0.76 (0.72-0.81) |
| 60-90 days | 1,490 | 0.83 (0.78-0.88) | 1,401 | 0.77 (0.73-0.82) | 1,469 | 0.80 (0.75-0.85) | 1,495 | 0.80 (0.75-0.85) |
| 91-120 days | 1,357 | 0.81 (0.76-0.86) | 1,478 | 0.87 (0.82-0.93) | 1,491 | 0.87 (0.82-0.92) | 1,499 | 0.86 (0.81-0.91) |
| Baseline | 11,052 | ref | 11,198 | ref | 11,479 | ref | 11,762 | ref |
| Risk period | 2016/17 | | 2017/18 | | 2018/19 | |  |  |
|  | N events | IR (95% CI) | N events | IR (95% CI) | N events | IR (95% CI) |  |  |
| 15-28 days | 628 | 0.66 (0.60-0.71) | 752 | 0.71 (0.66-0.77) | 713 | 0.73 (0.67-0.79) |  |  |
| 29-59 days | 1,563 | 0.76 (0.71-0.80) | 1,652 | 0.72 (0.68-0.76) | 1,599 | 0.75 (0.71-0.80) |  |  |
| 60-90 days | 1,524 | 0.76 (0.72-0.81) | 1,667 | 0.76 (0.72-0.80) | 1,570 | 0.76 (0.72-0.81) |  |  |
| 91-120 days | 1,481 | 0.79 (0.75-0.84) | 1,663 | 0.81 (0.77-0.86) | 1,629 | 0.85 (0.80-0.90) |  |  |
| Baseline | 12,409 | ref | 13,545 | ref | 13,086 | ref |  |  |

Supplementary Table 4. Incidence ratios for first acute cardiovascular event in the 14 days after influenza vaccination

| Time period | All | | QRISK2 | | | | Hypertension | | | |
| --- | --- | --- | --- | --- | --- | --- | --- | --- | --- | --- |
|  |  |  | Raised risk | | Low risk | | Raised risk | | Low risk | |
|  | N events | IR (95% CI) | N events | IR (95% CI) | N events | IR (95% CI) | N events | IR (95% CI) | N events | IR (95% CI) |
| 1-7 days | 3,370 | 0.68 (0.66-0.71) | 3,048 | 0.72 (0.69-0.75) | 322 | 0.48 (0.43-0.53) | 1,830 | 0.71 (0.68-0.74) | 1,540 | 0.66 (0.62-0.69) |
| 8-14 days | 3,495 | 0.71 (0.68-0.73) | 3,160 | 0.75 (0.72-0.78) | 335 | 0.49 (0.44-0.55) | 1,946 | 0.75 (0.72-0.79) | 1,549 | 0.66 (0.62-0.69) |

Supplementary Table 5. Incidence ratios for non-fatal first acute cardiovascular event in risk periods following influenza vaccination among individuals by cardiovascular risk and age group

| Risk period | All | | QRISK2 | | | | Hypertension | | | |
| --- | --- | --- | --- | --- | --- | --- | --- | --- | --- | --- |
|  |  |  | Raised risk | | Low risk | | Raised risk | | Low risk | |
|  | N events | IR (95% CI) | N events | IR (95% CI) | N events | IR (95% CI) | N events | IR (95% CI) | N events | IR (95% CI) |
| All ages |  |  |  |  |  |  |  |  |  |  |
| 15-28 days | 6,541 | 0.76 (0.74-0.78) | 5,916 | 0.81 (0.79-0.84) | 625 | 0.49 (0.45-0.53) | 3,638 | 0.82 (0.79-0.85) | 2,903 | 0.70 (0.67-0.72) |
| 29-59 days | 14,634 | 0.77 (0.76-0.79) | 13,261 | 0.83 (0.81-0.85) | 1,373 | 0.49 (0.46-0.52) | 8,130 | 0.84 (0.81-0.86) | 6,504 | 0.71 (0.69-0.73) |
| 60-90 days | 14,735 | 0.79 (0.77-0.80) | 13,389 | 0.85 (0.83-0.87) | 1,346 | 0.49 (0.46-0.52) | 8,236 | 0.86 (0.84-0.88) | 6,499 | 0.72 (0.70-0.74) |
| 91-120 days | 14,491 | 0.82 (0.80-0.83) | 13,162 | 0.88 (0.86-0.89) | 1,329 | 0.52 (0.49-0.55) | 8,036 | 0.88 (0.86-0.90) | 6,455 | 0.75 (0.73-0.78) |
| Baseline | 118,508 | ref | 103,929 | ref | 14,579 | ref | 63,880 | ref | 54,628 | ref |
| 40-64 years |  |  |  |  |  |  |  |  |  |  |
| 15-28 days | 1,336 | 0.55 (0.52-0.58) | 795 | 0.63 (0.58-0.68) | 541 | 0.46 (0.42-0.50) | 567 | 0.64 (0.59-0.70) | 769 | 0.49 (0.46-0.53) |
| 29-59 days | 2,937 | 0.55 (0.53-0.57) | 1,742 | 0.63 (0.59-0.66) | 1,195 | 0.46 (0.44-0.49) | 1,290 | 0.67 (0.63-0.71) | 1,647 | 0.48 (0.46-0.51) |
| 60-90 days | 2,878 | 0.55 (0.53-0.57) | 1,706 | 0.63 (0.59-0.66) | 1,172 | 0.47 (0.44-0.50) | 1,269 | 0.67 (0.63-0.71) | 1,609 | 0.49 (0.46-0.51) |
| 91-120 days | 2,859 | 0.59 (0.56-0.61) | 1,705 | 0.67 (0.63-0.70) | 1,154 | 0.50 (0.47-0.53) | 1,270 | 0.71 (0.66-0.75) | 1,589 | 0.52 (0.49-0.55) |
| Baseline | 29,067 | ref | 16,091 | ref | 12,976 | ref | 11,571 | ref | 17,496 | ref |
| 65-74 years |  |  |  |  |  |  |  |  |  |  |
| 15-28 days | 2,347 | 0.84 (0.80-0.88) | 2,263 | 0.84 (0.80-0.88) | 84 | 0.81 (0.64-1.02) | 1,254 | 0.86 (0.81-0.91) | 1,093 | 0.82 (0.77-0.87) |
| 29-59 days | 5,287 | 0.86 (0.83-0.88) | 5,109 | 0.86 (0.83-0.89) | 178 | 0.77 (0.65-0.92) | 2,759 | 0.86 (0.82-0.89) | 2,528 | 0.86 (0.82-0.90) |
| 60-90 days | 5,388 | 0.88 (0.85-0.91) | 5,214 | 0.89 (0.86-0.91) | 174 | 0.76 (0.64-0.90) | 2,850 | 0.89 (0.85-0.93) | 2,538 | 0.87 (0.83-0.91) |
| 91-120 days | 5,358 | 0.92 (0.89-0.95) | 5,183 | 0.92 (0.89-0.95) | 175 | 0.79 (0.67-0.94) | 2,809 | 0.92 (0.88-0.96) | 2,549 | 0.91 (0.87-0.95) |
| Baseline | 42,262 | ref | 40,659 | ref | 1,603 | ref | 22,386 | ref | 19,876 | ref |
| 75-84 years |  |  |  |  |  |  |  |  |  |  |
| 15-28 days | 2,858 | 0.88 (0.84-0.92) |  |  |  |  | 1,817 | 0.89 (0.84-0.93) | 1,041 | 0.87 (0.81-0.93) |
| 29-59 days | 6,410 | 0.90 (0.87-0.92) |  |  |  |  | 4,081 | 0.91 (0.87-0.94) | 2,329 | 0.88 (0.84-0.93) |
| 60-90 days | 6,469 | 0.92 (0.89-0.95) |  |  |  |  | 4,117 | 0.93 (0.89-0.96) | 2,352 | 0.90 (0.86-0.95) |
| 91-120 days | 6,274 | 0.94 (0.91-0.97) |  |  |  |  | 3,957 | 0.94 (0.90-0.98) | 2,317 | 0.94 (0.89-0.98) |
| Baseline | 47,179 | ref |  |  |  |  | 29,923 | ref | 17,256 | ref |

QRISK2 score results are not included for those aged 75-84 years as all individuals were high risk.

Supplementary Table 6. Incidence ratios for first acute cardiovascular event in risk periods following influenza vaccination by further QRISK2 stratification

| Risk period | QRISK2 | | | | | |
| --- | --- | --- | --- | --- | --- | --- |
|  | ≥20% | | 10-19% | | <10% | |
|  | N events | IR (95% CI) | N events | IR (95% CI) | N events | IR (95% CI) |
| All ages |  |  |  |  |  |  |
| 15-28 days | 4,756 | 0.79 (0.77-0.82) | 1,671 | 0.71 (0.67-0.75) | 657 | 0.83 (0.67-1.02) |
| 29-59 days | 10,912 | 0.85 (0.83-0.87) | 3,655 | 0.72 (0.69-0.74) | 1,466 | 0.78 (0.67-0.92) |
| 60-90 days | 11,107 | 0.90 (0.88-0.92) | 3,671 | 0.74 (0.71-0.77) | 1,431 | 0.86 (0.73-1.00) |
| 91-120 days | 10,875 | 0.95 (0.93-0.97) | 3,590 | 0.77 (0.75-0.80) | 1,433 | 0.87 (0.75-1.03) |
| Baseline | 80,450 | ref | 30,841 | ref | 15,082 | ref |
| 40-64 |  |  |  |  |  |  |
| 15-28 days | 310 | 0.76 (0.67-0.86) | 527 | 0.57 (0.52-0.62) | 565 | 0.45 (0.41-0.49) |
| 29-59 days | 690 | 0.78 (0.71-0.85) | 1,155 | 0.57 (0.53-0.61) | 1,276 | 0.47 (0.44-0.50) |
| 60-90 days | 679 | 0.79 (0.72-0.86) | 1,131 | 0.58 (0.54-0.62) | 1,247 | 0.48 (0.45-0.51) |
| 91-120 days | 676 | 0.83 (0.76-0.91) | 1,114 | 0.62 (0.58-0.66) | 1,244 | 0.52 (0.49-0.56) |
| Baseline | 5,615 | ref | 11,013 | ref | 13,394 | ref |
| 65-74 |  |  |  |  |  |  |
| 15-28 days | 1,387 | 0.79 (0.74-0.83) | 1,038 | 0.82 (0.77-0.88) | 92 | 0.80 (0.64-1.00) |
| 29-59 days | 3,225 | 0.85 (0.81-0.88) | 2,284 | 0.83 (0.79-0.88) | 190 | 0.76 (0.64-0.90) |
| 60-90 days | 3,368 | 0.91 (0.88-0.95) | 2,313 | 0.87 (0.83-0.91) | 184 | 0.75 (0.64-0.89) |
| 91-120 days | 3,320 | 0.96 (0.92-1.00) | 2,260 | 0.90 (0.86-0.95) | 189 | 0.82 (0.69-0.96) |
| Baseline | 24,915 | ref | 18,105 | ref | 1,688 | ref |
| 75-84 |  |  |  |  |  |  |
| 15-28 days | 3,059 | 0.80 (0.77-0.83) | 106 | 0.83 (0.67-1.02) |  |  |
| 29-59 days | 6,997 | 0.86 (0.83-0.88) | 216 | 0.78 (0.67-0.92) |  |  |
| 60-90 days | 7,060 | 0.91 (0.88-0.94) | 227 | 0.86 (0.73-1.00) |  |  |
| 91-120 days | 6,879 | 0.96 (0.93-0.99) | 216 | 0.87 (0.75-1.03) |  |  |
| Baseline | 49,920 | ref | 1,723 | ref |  |  |

QRISK2 score <10% results are not included for those aged 75-84 years as all individuals had a score ≥10%Supplementary Table 7. Baseline characteristics of sensitivity analysis study design population

|  | All | QRISK2 | | Hypertension | |
| --- | --- | --- | --- | --- | --- |
|  |  | Raised risk | Low risk | Raised risk | Low risk |
|  | n=160,906 | n=147,023 | n=13,883 | n=90,992 | n=69,914 |
| Sex |  |  |  |  |  |
| Female (%) | 77,467 (48.1%) | 67,783 (46.1%) | 9,684 (69.8%) | 45,747 (50.3%) | 31,720 (45.4%) |
| Age group (years) |  |  |  |  |  |
| 40-64 | 29,927 (18.6%) | 18,127 (12.3%) | 11,800 (85.0%) | 13,523 (14.9%) | 16,404 (23.5%) |
| 65-74 | 59,168 (36.8%) | 57,085 (38.8%) | 2,083 (15.0%) | 31,631 (34.8%) | 27,537 (39.4%) |
| 75-84 | 71,811 (44.6%) | 71,811 (48.8%) | 0 (0.0%) | 45,838 (50.4%) | 25,973 (37.1%) |
| Associated hospital stay |  |  |  |  |  |
| Yes | 112,994 (70.2%) | 103,455 (70.4%) | 9,539 (68.7%) | 64,192 (70.5%) | 48,802 (69.8%) |
| Median (IQR) stay | 4.0 (2.0-11.0) | 4.0 (2.0-11.0) | 3.0 (1.0-8.0) | 4.0 (2.0-11.0) | 4.0 (2.0-10.0) |
| Died ≤30 days after event |  |  |  |  |  |
| Yes | 13,098 (8.1%) | 12,260 (8.3%) | 838 (6.0%) | 7,584 (8.3%) | 5,514 (7.9%) |
| Died in study period |  |  |  |  |  |
| Yes | 18,644 (11.6%) | 17,525 (11.9%) | 1,119 (8.1%) | 10,853 (11.9%) | 7,791 (11.1%) |

Supplementary Table 8. Sensitivity analysis study design incidence ratios for first acute cardiovascular event in risk periods following influenza vaccination by cardiovascular risk and age group

| Risk period | All | | QRISK2 | | | | Hypertension | | | |
| --- | --- | --- | --- | --- | --- | --- | --- | --- | --- | --- |
|  |  |  | Raised risk | | Low risk | | Raised risk | | Low risk | |
|  | N events | IR (95% CI) | N events | IR (95% CI) | N events | IR (95% CI) | N events | IR (95% CI) | N events | IR (95% CI) |
| All ages |  |  |  |  |  |  |  |  |  |  |
| 15-28 days | 6,923 | 0.94 (0.91-0.96) | 6,313 | 0.94 (0.91-0.96) | 610 | 0.95 (0.86-1.04) | 3,885 | 0.93 (0.90-0.97) | 3,038 | 0.95 (0.91-0.99) |
| 29-59 days | 15,752 | 0.96 (0.95-0.98) | 14,356 | 0.96 (0.94-0.98) | 1,396 | 0.98 (0.91-1.05) | 8,797 | 0.95 (0.93-0.98) | 6,955 | 0.98 (0.95-1.01) |
| 60-90 days | 15,999 | 0.98 (0.96-1.00) | 14,637 | 0.98 (0.96-1.00) | 1,362 | 0.96 (0.89-1.02) | 9,032 | 0.98 (0.95-1.00) | 6,967 | 0.98 (0.95-1.01) |
| 91-120 days | 15,740 | 1.00 (0.98-1.02) | 14,363 | 1.00 (0.98-1.02) | 1,377 | 1.00 (0.93-1.07) | 8,819 | 0.99 (0.96-1.01) | 6,921 | 1.01 (0.98-1.04) |
| Baseline | 99,885 | ref | 91,346 | ref | 8,539 | ref | 56,780 | ref | 43,105 | ref |
| 40-64 years |  |  |  |  |  |  |  |  |  |  |
| 15-28 days | 1,353 | 1.01 (0.95-1.08) | 827 | 1.04 (0.96-1.13) | 526 | 0.97 (0.88-1.07) | 585 | 0.98 (0.90-1.08) | 768 | 1.03 (0.95-1.12) |
| 29-59 days | 3,036 | 1.03 (0.98-1.08) | 1,821 | 1.03 (0.97-1.10) | 1,215 | 1.01 (0.94-1.09) | 1,357 | 1.03 (0.96-1.11) | 1,679 | 1.02 (0.96-1.09) |
| 60-90 days | 2,989 | 1.01 (0.96-1.06) | 1,804 | 1.02 (0.96-1.09) | 1,185 | 0.99 (0.92-1.06) | 1,327 | 1.01 (0.94-1.08) | 1,662 | 1.01 (0.95-1.08) |
| 91-120 days | 2,986 | 1.04 (0.99-1.09) | 1,791 | 1.05 (0.99-1.11) | 1,195 | 1.03 (0.96-1.11) | 1,336 | 1.04 (0.97-1.12) | 1,650 | 1.04 (0.97-1.10) |
| Baseline | 18,211 | ref | 11,055 | ref | 7,156 | ref | 8,300 | ref | 9,911 | ref |
| 65-74 years |  |  |  |  |  |  |  |  |  |  |
| 15-28 days | 2,470 | 0.91 (0.87-0.95) | 2,386 | 0.92 (0.87-0.96) | 84 | 0.83 (0.65-1.06) | 1,328 | 0.92 (0.86-0.98) | 1,142 | 0.91 (0.85-0.97) |
| 29-59 days | 5,605 | 0.93 (0.90-0.97) | 5,424 | 0.94 (0.91-0.97) | 181 | 0.81 (0.67-0.97) | 2,934 | 0.92 (0.87-0.96) | 2,671 | 0.96 (0.91-1.01) |
| 60-90 days | 5,789 | 0.97 (0.93-1.00) | 5,612 | 0.97 (0.94-1.01) | 177 | 0.79 (0.66-0.95) | 3,088 | 0.96 (0.92-1.01) | 2,701 | 0.97 (0.92-1.02) |
| 91-120 days | 5,715 | 0.99 (0.95-1.02) | 5,533 | 0.99 (0.96-1.02) | 182 | 0.84 (0.70-1.01) | 3,014 | 0.97 (0.93-1.02) | 2,701 | 1.00 (0.95-1.05) |
| Baseline | 37,202 | ref | 35,819 | ref | 1,383 | ref | 20,007 | ref | 17,195 | ref |
| 75-84 years |  |  |  |  |  |  |  |  |  |  |
| 15-28 days | 3,100 | 0.93 (0.90-0.97) |  |  |  |  | 1,972 | 0.93 (0.88-0.98) | 1,128 | 0.94 (0.88-1.01) |
| 29-59 days | 7,111 | 0.97 (0.94-1.00) |  |  |  |  | 4,506 | 0.96 (0.92-0.99) | 2,605 | 0.98 (0.93-1.03) |
| 60-90 days | 7,221 | 0.98 (0.95-1.01) |  |  |  |  | 4,617 | 0.98 (0.95-1.02) | 2,604 | 0.98 (0.93-1.03) |
| 91-120 days | 7,039 | 0.99 (0.96-1.02) |  |  |  |  | 4,469 | 0.98 (0.95-1.02) | 2,570 | 1.00 (0.95-1.05) |
| Baseline | 44,472 | ref |  |  |  |  | 28,473 | ref | 15,999 | ref |

QRISK2 score results are not included for those aged 75-84 years as all individuals were high risk.


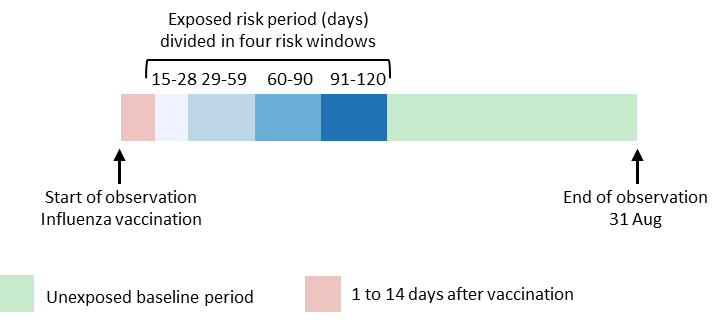


Supplementary Figure 1. Overview of sensitivity analysis study design


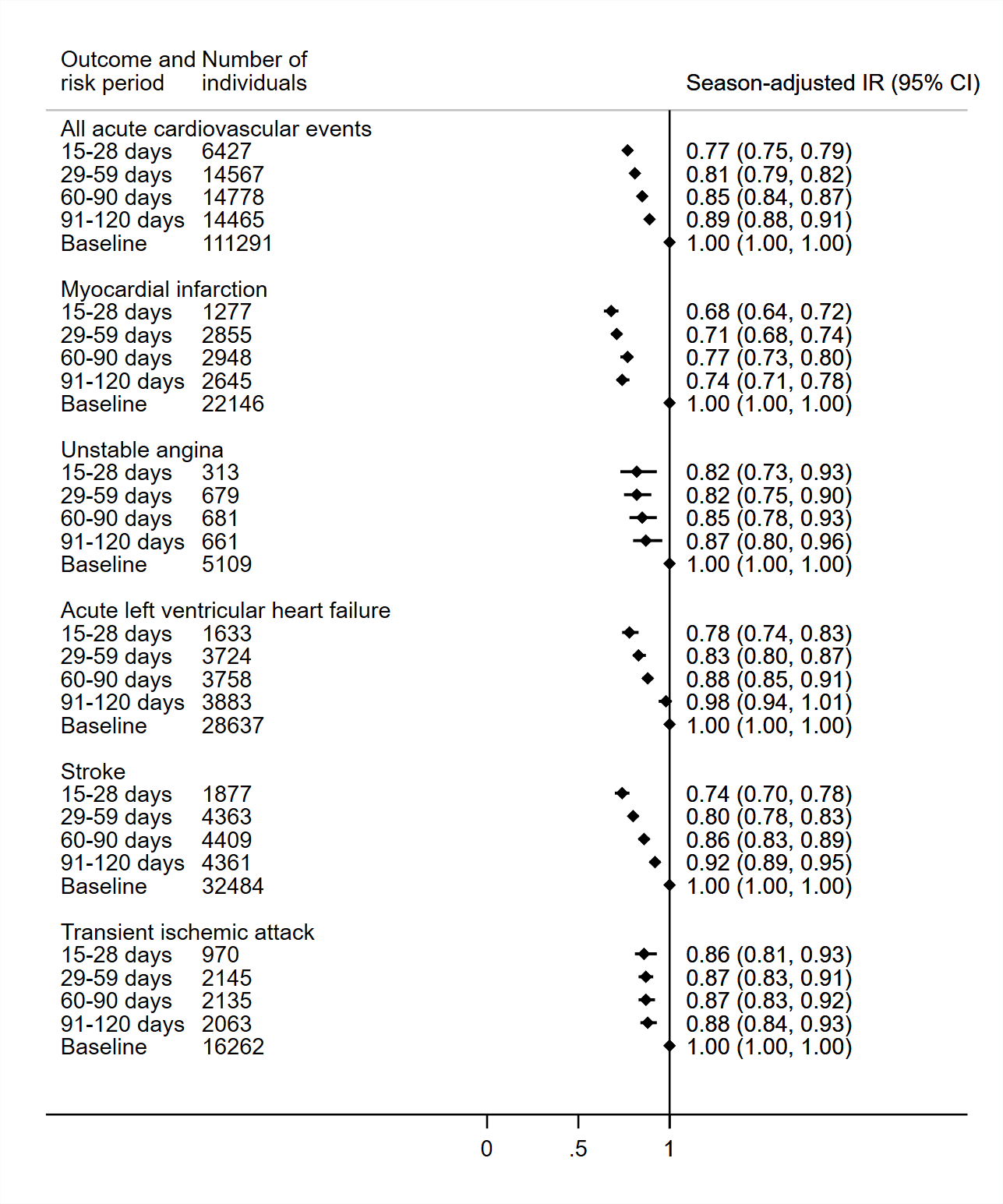


Supplementary Figure 2. Incidence ratios for first acute cardiovascular events in risk periods following influenza vaccination among individuals with a QRISK2 score ≥10% by cardiovascular event type


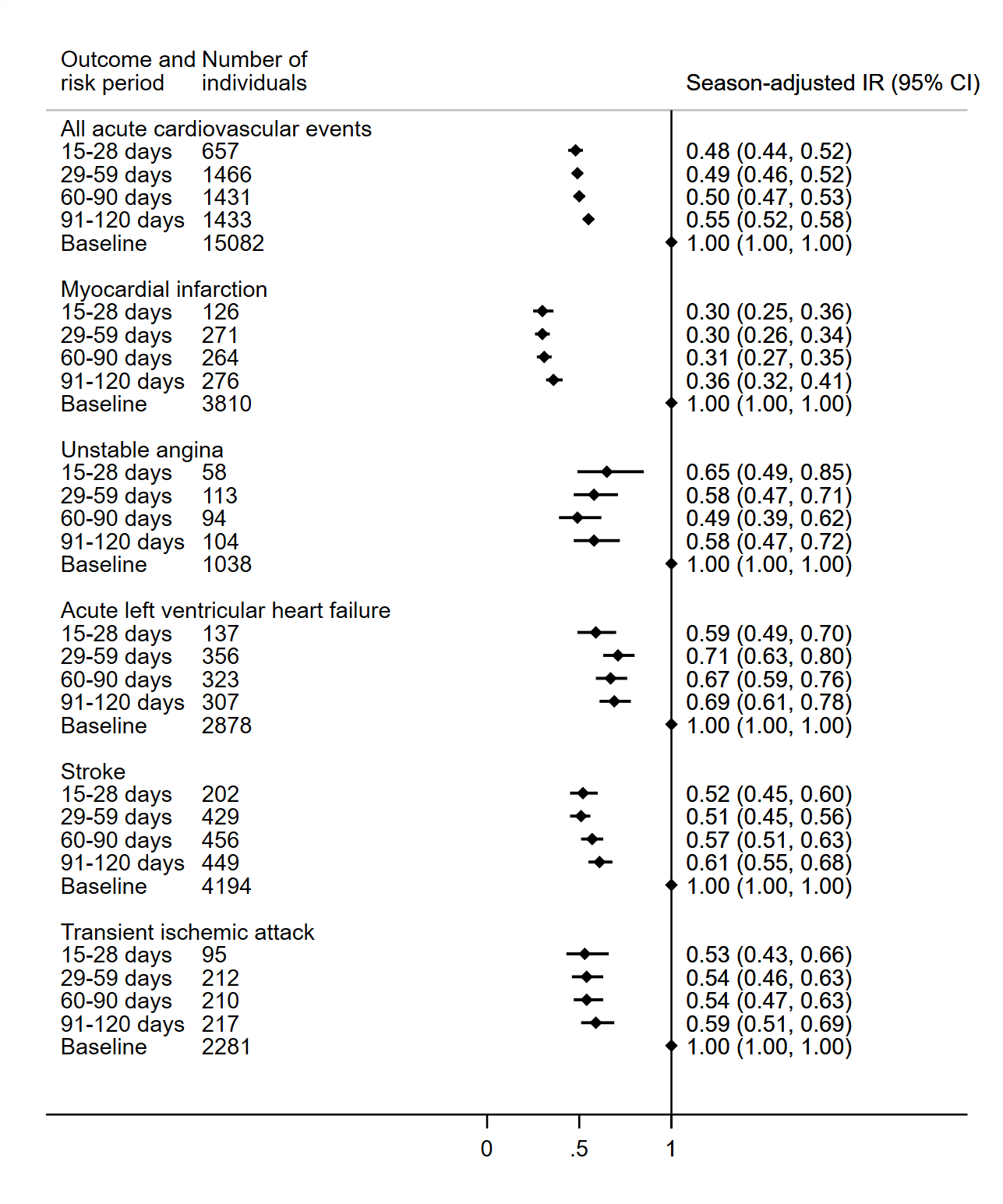


Supplementary Figure 3. Incidence ratios for first acute cardiovascular events in risk periods following influenza vaccination among individuals with a QRISK2 score <10% by cardiovascular event type


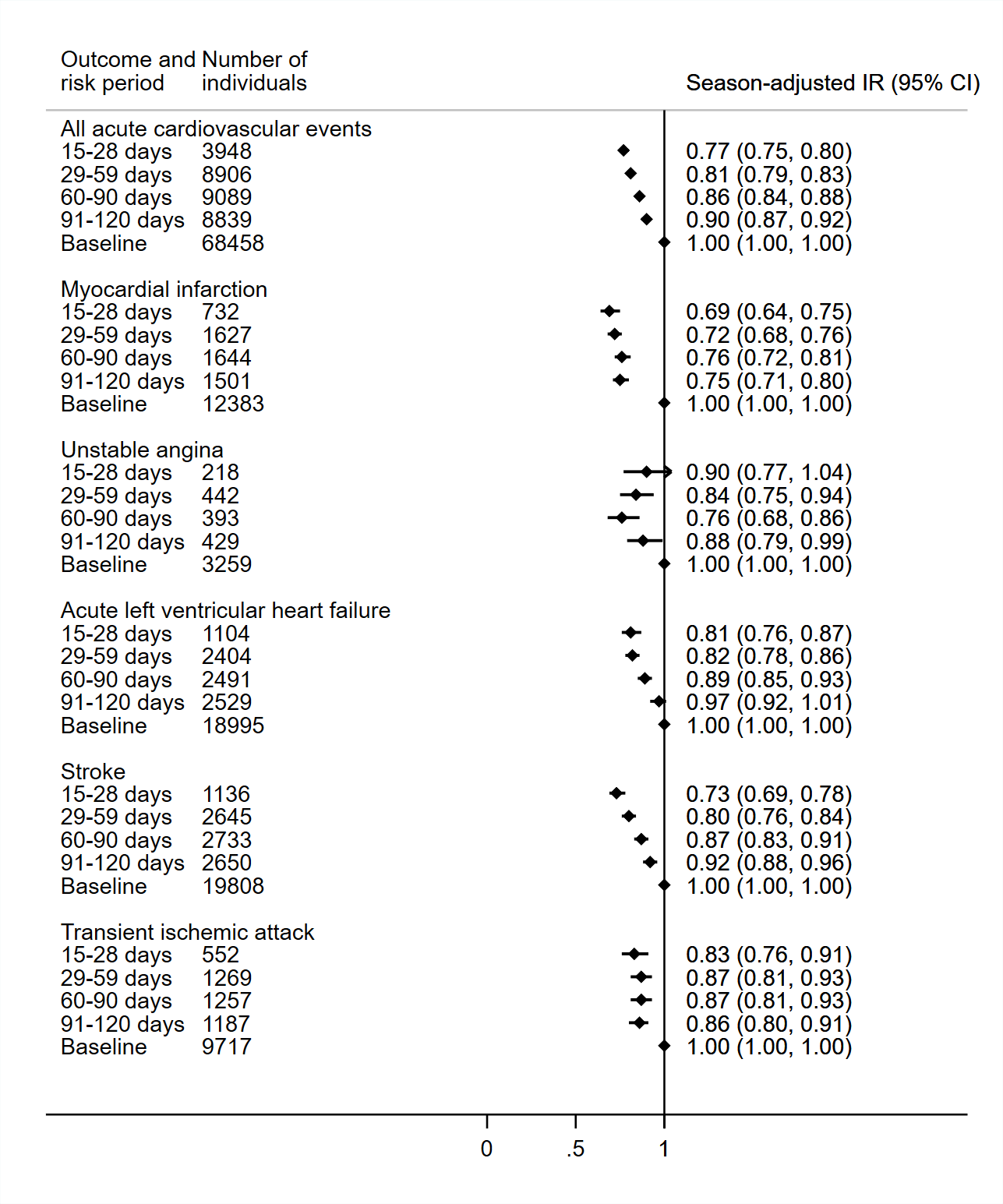


Supplementary Figure 4. Incidence ratios for first acute cardiovascular events in risk periods following influenza vaccination among individuals with diagnosed hypertension by cardiovascular event type


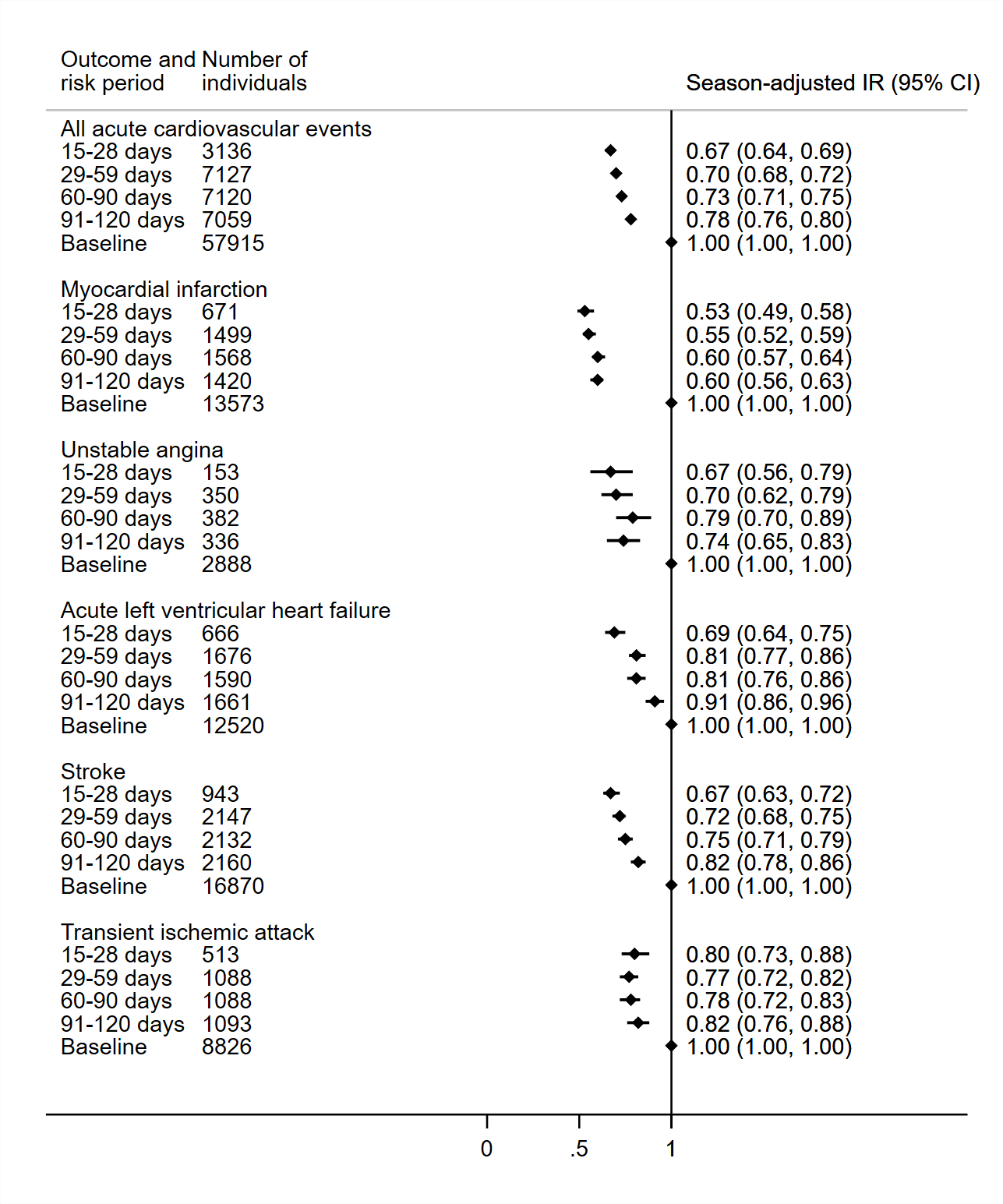


Supplementary Figure 5. Incidence ratios for first acute cardiovascular events in risk periods following influenza vaccination among individuals without diagnosed hypertension by cardiovascular event type


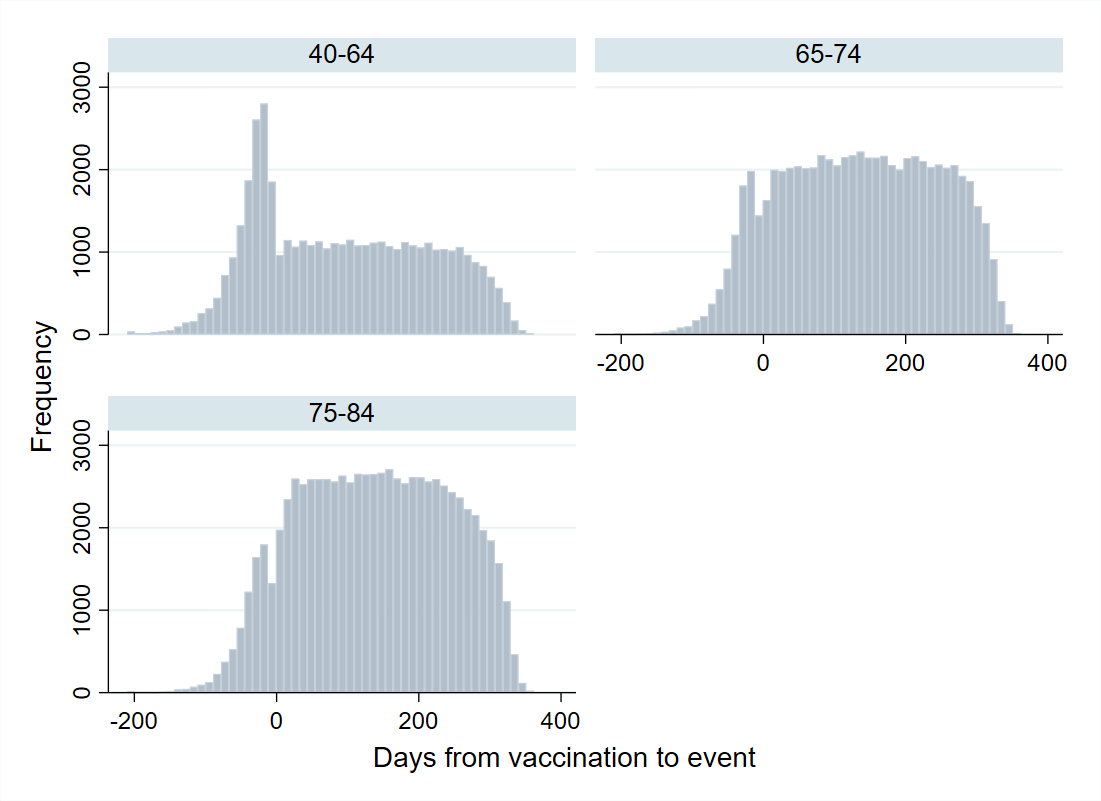


Supplementary Figure 6. Difference in time (days) between vaccination and acute cardiovascular event by age group
